# DegNorm: normalization of generalized transcript degradation improves accuracy in RNA-seq analysis

**DOI:** 10.1186/s13059-019-1682-7

**Published:** 2019-04-16

**Authors:** Bin Xiong, Yiben Yang, Frank R. Fineis, Ji-Ping Wang

**Affiliations:** Department of Statistics, Northwestern University, Evanston, IL 60208 USA

**Keywords:** RNA-seq, Normalization, RNA degradation, Degradation normalization, Alternative splicing, Non-negative matrix factorization

## Abstract

RNA degradation affects RNA-seq quality when profiling transcriptional activities in cells. Here, we show that transcript degradation is both gene- and sample-specific and is a common and significant factor that may bias the results in RNA-seq analysis. Most existing global normalization approaches are ineffective to correct for degradation bias. We propose a novel pipeline named DegNorm to adjust the read counts for transcript degradation heterogeneity on a gene-by-gene basis while simultaneously controlling for the sequencing depth. The robust and effective performance of this method is demonstrated in an extensive set of simulated and real RNA-seq data.

**Electronic supplementary material:**

The online version of this article (10.1186/s13059-019-1682-7) contains supplementary material, which is available to authorized users.

## Background

RNA-seq is currently the most prevailing method for profiling transcriptional activities using high-throughput sequencing technology [1]. The sequencing tag count per unit of transcript length is used to measure the relative abundance of the transcript [2]. Various factors exist that may affect the faithful representation of transcript abundance by RNA-seq read counts. Normalization is a crucial step in post-experiment data processing to ensure a fair comparison of gene expression in RNA-seq analysis [3, 4]. The most commonly used approach is to normalize the read counts globally by a sample-specific scale factor to adjust the sequencing depth. Choices of the scale factor include the total number of reads (or mean), median, trimmed mean of *M* values [5], and upper quartile [3]. The second type of normalization aims to remove the read count bias due to physical or chemical features of RNA sequences or uncontrollable technical aspects. The GC content is known to affect the read counts in a nonlinear way [6, 7], and this effect can be sample specific under different culture or library preparation protocols [8]. Systematic bias may also arise due to technical effects such as library preparation and sequencing batches. Such systematic biases can be quantified and removed using factor analysis provided that the unwanted variation is uncorrelated with the covariates of interest [9, 10].

Another type of bias arises from cDNA fragmentation and mRNA degradation. The RNA-seq assay requires fragmenting the cDNA (reversely transcribed from mRNA) or mRNA for high-throughput sequencing. Ideally, for a complete, non-degraded transcript, if the fragmentation is completely random, we expect to see reads uniformly distributed along the transcript. Nevertheless, the fragmentation by random priming is not truly random due to primer specificity [11–13]. Consequently, read count per unit length of a transcript may not strictly reflect the transcript abundance when comparing the expression of different genes. For the same gene, assuming the same protocol is applied to different samples, the bias attributable to fragmentation across samples should be similar. Thus, fragmentation bias is less problematic in a gene-by-gene differential expression (DE) analysis. In contrast, mRNA degradation can vary substantially in both extent and pattern between genes and between samples [14, 15]. The mRNA degradation has different pathways and can happen in any region of a transcript [16]. Perfect control of sample degradation during the experiment is difficult, particularly when the samples are collected from field studies or clinical samples. More importantly, different genes may degrade at different rates [17], which makes it impossible to remove this bias by normalizing the read counts of all genes in the same sample by the same constant.

While the major impact of RNA degradation on gene expression analysis has been well recognized [17, 18], methods for correcting the degradation bias have not been fully explored in the literature. A few methods have been proposed to quantify the RNA integrity including RNA integrity numbers (RIN) [19], mRIN [20], and transcript integrity number (TIN) [13]. The RIN gives a sample-specific overall RNA quality measure, but not at the gene level. In practice, a sample with RIN ≥ 7 (on a scale of 0 to 10) is often regarded as having good quality. The mRIN and TIN measures were both defined in the gene level by comparing the sample read distribution with reference to the hypothetical uniform distribution. In real data, due to GC content bias, primer specificity, and other complexities, the read count may substantially deviate from the uniform distribution along the transcript [7].

To reduce the degradation effect, Finotello et al. proposed to quantify the exon-level expression by the maximum of its per-base counts instead of the raw read counts [21]. If a given exon is in a more degraded region, the local maximum may still be an underestimate of true abundance. On the other hand, the larger variance associated with the local maximum (e.g., spikes) may result in instability in DE analysis. Based on the TIN measure, Wang et al. proposed a degradation normalization method based on loess regression of read counts on the TIN measure for genes within the same sample [13]. However, the uniform baseline assumption and the failure to compare gene-specific degradation across samples appear to be the two major limitations, which may lead to extreme variability and bias in DE analysis (to be shown below). Jaffe et al. proposed a quality surrogate variable analysis (qSVA) to remove the confounding effect of RNA quality in DE analysis [22]. They investigated the degradation of RNA-seq data from dorsolateral prefrontal cortex (DLPFC) tissue under two different RNA-seq protocols, namely, poly(A)+ (mRNA-seq) vs. ribosomal depletion (Ribo-Zero-seq). Thousands of features significantly associated with degradation were identified under either protocol separately, while no overlap was found between the two protocols. Furthermore, comparing the DLPFC samples and the peripheral blood mononuclear cell (PBMC) samples [17] both sequenced under the same poly(A)+ protocol, they found only four shared features. It is unclear how the sequence features identified in this study can be generalized for degradation bias correction in other RNA-seq data in practice.

Alternative splicing is frequently observed in higher organisms, and it further complicates the gene expression estimation in RNA-seq [23]. In the gene-level DE analysis, we test the equivalence of relative abundance of transcripts in copy numbers between samples or conditions. If the two samples have differential exon usage, read counts need to be adjusted accordingly to better represent the transcript relative abundance in the respective samples. Currently, most existing statistical packages for RNA-seq analysis (e.g., DESeq [24] and edgeR [25]) all take the raw read counts as input, while such complexities are completely ignored in practice.

In this paper, we propose a novel data-driven method to quantify the transcript degradation in a generalized sense for each gene within each sample. Using the estimated degradation index scores, we build a normalization pipeline named DegNorm to correct for degradation bias on a gene-by-gene basis while simultaneously controlling the sequencing depth. The performance of the proposed pipeline is investigated using simulated data, and an extensive set of real data that came from both cell line and clinical samples sequenced in poly(A)+ or Ribo-Zero protocol.

## Results

### Data sets

We consider six RNA-seq data sets that were generated from cell lines or clinical samples under either the mainstream poly(A) enrichment (mRNA-seq) or ribosomal RNA depletion protocol (Ribo-Zero-seq). The first one was from a brain glioblastoma (GBM) cell line study of a human for the impact of RNA degradation on gene expression analysis [26]. Technical replicates of RNA samples were fragmented under different incubation time and temperature using the NEBNext Magnesium RNA fragmentation module. We chose to analyze nine mRNA-seq samples in three groups of three, corresponding to three average RNA integrity number (RIN) = 10, 6, and 4, respectively (to be referred to as R10, R6, and R4 for simplicity). We will perform DE analysis for R10 vs. R4 and R6 vs. R4.

The second set contained 32 single-end mRNA-seq samples from human peripheral blood mononuclear cells (PBMC) of 4 different subjects: S00, S01, S02, and S03 [17]. The extracted RNA sample from each subject was kept in room temperature for 0, 12, 24, 36, 48, 60, 72, and 84 h, respectively, to approximate the natural degradation process. We choose S01 as an illustrating example and will perform DE analysis for 0 + 12 h vs. 24 + 48 h (results for other subjects are similar).

The third set was from Sequencing Quality Control (SEQC) Consortium [27, 28] and contained two subsets of mRNA-seq data, namely SEQC-AA and SEQC-AB. The SEQC-AA subset consisted of 16 technical replicates from Stratagene’s universal human reference (UHR) RNA library with two runs for eight lanes each. We will run DE analysis of the first run vs. the second run. The second subset contained two biological conditions: condition A of five samples from the same Stratagene’s UHR RNA, and condition B of five samples from Ambion’s human brain reference RNA. The first four replicates from both conditions were prepared by the same technician while the fifth was by Illumina. We excluded the fifth sample from both conditions because they showed a dramatic difference in coverage curves compared to the rest.

The fourth data set contained RNA-seq data from dorsolateral prefrontal cortex (DLPFC) tissue of five brains—three controls and two schizophrenia cases [22]. Each tissue was left in room temperature (off of ice) for 0, 15, 30, and 60 min for degradation. The RNA sample was extracted and prepared for both mRNA-seq and Ribo-Zero-seq. We chose to analyze one schizophrenia case (Br1729, results for other subjects are similar). We will perform DE analysis T0+T15 min vs. T30+T60 min under the same protocol, i.e., mRNA-seq or Ribo-Zero-seq, and then cross-platform DE analysis between the two protocols.

The fifth data set originated from three pairs of matched fresh-frozen (FF) and formalin-fixed paraffin-embedded (FFPE) tissues of three breast tumor patients (namely T1, T2, T3) with a moderate archival time of about 4–5 years [29]. The FFPE samples are typically partially degraded. We will analyze the mRNA-seq data of FF (500 ng) and FFPE (100 ng) to investigate whether the degradation normalization can help improve the DE analysis in fragmented clinical RNA samples.

The last data set arose from a clinical study on how AMP kinase (AMPK) promotes glioblastoma bioenergetics and tumor growth [30]. It was shown that cancer cells can activate AMPK and highjack the stress-regulating pathway in cells. Thus, inhibiting AMPK in cancer cells may lead to treatment of GBM. RNA-seq data was collected from two patient-derived GBM stem cell (GSC) lines (GBM9 and GBM10) between control and AMPK knockout to identify differentially expressed genes. We will perform DE analysis between the three control and three knockout samples in GBM10 cell line. Unlike the first five sets where the ground truth of gene expression was known or mostly verified, the AMPK knockout data represents a typical case in clinical studies where only a handful genes of interest, most often suspected as differentially expressed, were PCR verified. For this reason, in the following analysis, we will compare the different normalization methods by benchmarking our analyses using the first five sets and then present the AMPK data as a case study in the last.

### Non-uniformity and heterogeneity in read distribution pattern

**Fig. 1:**
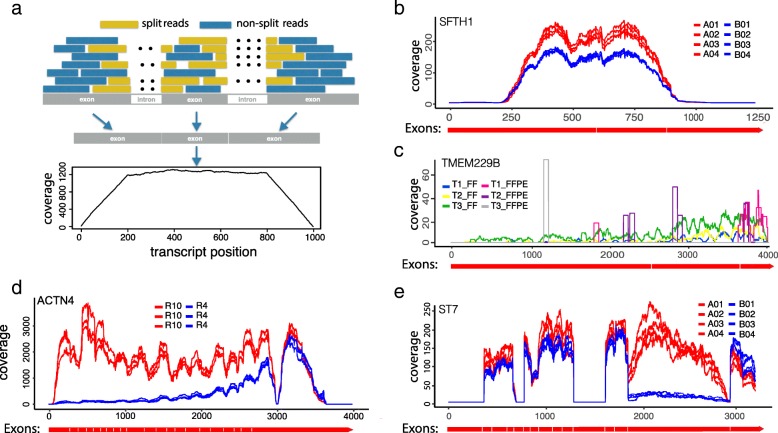
RNA-seq read coverage score shows between-sample heterogeneity in the pattern along transcripts. **a** The read coverage score, defined as the number of reads that cover each base pair, is expected to have a trapezoidal shape along the transcript if the read start position is uniformly distributed. **b** An example from SEQC-AB data shows a non-uniform but consistent read coverage pattern, where the average magnitude of coverage score for each sample may faithfully represent the transcript abundance given the sequencing depth is normalized. The diagram in red under the coverage plot shows the total transcript with exon boundaries from genome annotations (same for **c**, **d** and **e** below). **c** The TMEM229B gene from the breast tumor data shows differential coverage score patterns between FF and FFPE samples. Reads from FF samples are continuously distributed across the entire transcript while those from FFPE samples are highly enriched in a few disjoint blocks or fragments. **d** The ACTN4 gene from the GBM data with RIN number = 10 vs. RIN = 4 shows clear degraded coverage score towards the 5′ end of the transcripts in the latter group. **e** An example from the SEQC-AB data shows that alternative splicing likely causes sharply decayed coverage score across the entire alternatively spliced exon region

We define a total transcript as the concatenation of all annotated exons from the same gene. The read coverage score at a given location within the transcript is defined as the total number of reads (single-end) or DNA fragments (paired-end) that cover this position (Additional file 1). If mRNA transcripts are complete and the fragmentation is random, we expect to see a flat coverage curve in the entire transcript except in the head and tail region (Fig. 1a). Nevertheless, in real data, the read coverage curves rarely display a uniform pattern; instead, dramatic and gene-specific differences are often observed across samples (Fig. 1b–e). The non-uniformity itself is less concerning as long as the coverage pattern is consistent across samples (Fig. 1b) such that the read counts can still faithfully represent the relative abundance of transcripts. In contrast, heterogeneous coverage patterns are often observed where some samples show significantly decayed read counts in some regions (Fig.1c–e). One major cause of this heterogeneity is mRNA degradation, which is clearly shown in the case of ACTN4 gene of R4 samples from the GBM data (Fig. 1d). Different sample preparation methods may lead to distinct read distributions. For example, FFPE samples may show a highly localized discrete read distribution pattern in contrast to a continuous distribution typically observed in FF samples (Fig. 1c). Alternative splicing may also result in depleted read count in the entire region of an exon, as exemplified in the ST7 gene of B samples (region 1900–2900 bp) from the SEQC-AB data (Fig. 1e). For the gene-level DE analysis, loss of read count due to such complexities needs to be compensated to ensure unbiased quantification of gene expression.

### Generalized degradation and degradation normalization algorithm

The degradation we target to normalize is defined in a generalized sense. Any systematic decay of read count in any region of a transcript in one or more samples compared to the rest in the same study is regarded as degradation. Clearly, mRNA degradation is one main cause, but alternative splicing and other factors may be the confounders that are difficult to deconvolute. To avoid confusion, in the following context, we will reserve the term “mRNA degradation” for the physical degradation of mRNA sequences, and “degradation” or “transcript degradation” for the generalized degradation without specification.

**Fig. 2:**
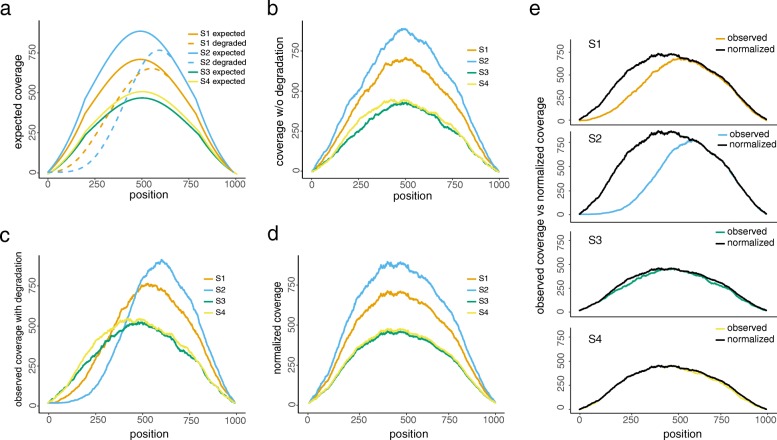
A proof-of-concept example of the proposed method for normalizing degradation patterns. **a** Expected or theoretical read coverage curves of one gene from four samples of identical shape (solid lines, without degradation), two of which (S1 and S2) are subject to degradation according to a rate indicated by the dashed lines in the 5′ end. **b** A realization of the four coverage curves without degradation randomly simulated according to the expected curves in **a**. These curves are regarded as the latent and unobserved data. **c** Observed coverage curves after imposing random degradation to S1 and S2 (S3 and S4 stay intact). **d** Estimates of the non-degraded latent curves from the proposed algorithm solely based on the observed coverage curves in **c**. **e** A sample-by-sample comparison between the observed and estimated latent coverage curves

We propose DegNorm, a degradation normalization pipeline based on non-negative matrix factorization over-approximation (NMF-OA, see the “Methods” section and Additional file 1). We assume there is a gene-specific ideal shape of coverage curve, called an “envelope” function, identical across the samples in the given study. Each envelope function is scaled by a sample- and gene-specific abundance parameter to represent the expected coverage curve for the given gene within each sample if no degradation occurs. Degradation may occur in any region of the transcript to cause negative bias in the observed read counts. To illustrate this, we generated four expected coverage curves of identical shape but with different abundance levels (Fig. 2a), among which samples S1 and S2 are subject to degradation in the 5′ end with different patterns. Based on the expected curves, we further simulated a random realization of four complete curves with sampling error imposed (Fig. 2b, to be referred to as latent curves) and two degraded for sample S1 and S2, respectively (Fig. 2c). The NMF-OA algorithm takes the four observed coverage curves (i.e., two non-degraded (S3 and S4) and two degraded (S1 and S2)) as input and estimates the latent curves by minimizing the squared distance between the observed and latent, subject to the constraint that the latent curves must dominate their respective observed curves at all positions (Figs. 2d, e; the “Methods” section). We define the degradation index (DI) score for each gene within each sample, as the fraction of area covered by the estimated latent curve, but above the observed curve (Fig. 2e). It measures the proportion of missing read count due to degradation given the current sequencing depth.

The DegNorm pipeline iteratively corrects for degradation bias while simultaneously normalizing sequencing depth. First, the NMF-OA algorithm is applied to sequencing depth-normalized read counts for all genes one by one to estimate the DI scores. Second, the resulting DI scores are then used to adjust the read counts by extrapolation for each gene. The adjusted read counts are used to normalize the raw data for sequencing depth. These two steps are repeated until the algorithm converges (the “Methods” section).

### DI score as sample quality diagnostics

**Fig. 3:**
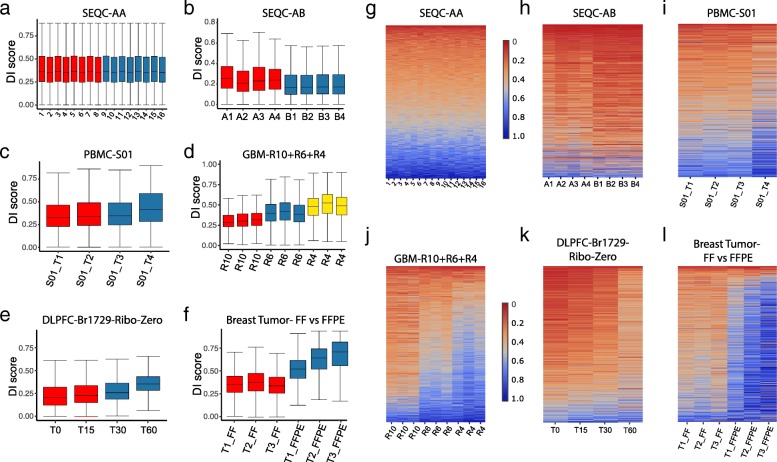
Degradation index (DI) scores show gene-/sample-/condition-specific degradation heterogeneity. **a**–**f** Box plots of DI scores presented in a between-group comparison defined for the differential expression analysis as follows. **a** SEQC-AA data (16,670 genes): the 8 (1–8) technical replicates from the first run vs. the 8 (9–16) from the second run. **b** SEQC-AB data (19,061 genes): 4 biological replicates from A condition vs. 4 from B condition. **c** PBMC data for subject S01 (14,051 genes): 2 samples exposed at room temperature for 0 and 12 h (S01_T1 and S01_T2) vs. 2 for 24 and 48 h (S01_T3 and S01_T4), respectively. **d** GBM data (14,298 genes): 3 replicates each for RIN number = 10, 6, and 4, respectively. **e** DLPFC data for subject Br1729 from Ribo-Zero-seq (18,634 genes): 2 samples exposed to room temperature for 0 and 15 min (T0, T15) vs. 2 for 30 and 60 min (T30, T60). **f** Breast tumor data of 3 matched pair (T1, T2, T3) prepared from FF and FFPE methods, respectively (10,996 genes). **g**–**l** For each data set presented in **a**–**f**, the heatmap presents the DI scores of genes sorted in the ascending order of the average DI score of the first condition (in the GBM case, the R10 samples), where each row corresponds to the same gene across samples

The estimated DI scores provide an overview of the within-sample, between-sample, and between-condition variation of degradation extent and patterns. We plotted the DI scores in three ways: a box plot of DI scores for each sample (Fig. 3a–f), a heatmap of the DI scores sorted in ascending order of the average scores of the first condition defined in the DE analysis (Fig. 3g–l), and a pairwise correlation matrix of DI scores between samples (Additional file 2: Figure S1a-f).

For SEQC-AA data with 16 technical replicates, the median of DI scores is ~ 0.35, consistent across samples (Fig. 3a). While the degradation pattern varies between genes, no systematic between-condition difference is observed (Fig. 3g, Additional file 2: Figure S1a). In contrast, for the SEQC-AB data, the 4 samples from condition B have relatively lower and more homogeneous degradation than that from A samples (Fig. 3b). Many genes show a condition-specific clustered pattern in DI scores (Fig. 3h), resulting in a high within-condition correlation (Additional file 2: Figure S1b).

The PBMC and GBM data are known to have differential mRNA degradation. The DI scores of PBMC S01 data confirm a progressive deterioration of average degradation when samples underwent degradation in room temperature for 0, 12, 24, and 48 h, respectively (Fig. 3c, i, Additional file 2: Figure S1c). The degradation from 24 to 48 h was particularly accelerated compared to the first 24 h. The nine GBM samples were previously classified into three groups according to the RNA integrity number (RIN), *R* = 10, 6, and 4, respectively. The DI scores show a clear escalating pattern of degradation severity across the three groups (Fig. 3d, j) with two strong clusters, i.e., R10 vs. R6+R4 (Additional file 2: Figure S1d). The scatter plots of DI scores further exemplify a higher correlation between samples within the same RIN group than across different RIN groups (Additional file 2: Figure S1 g-i).

For the DLPFC Br1729 Ribo-Zero-seq data, DegNorm recovered an increasing pattern of degradation from time 0 to 60 min as expected (Fig. 3e, k, Additional file 2: Figure S1e). The three pairs of breast tumor samples were prepared in two different ways—fresh frozen (FF) and formalin-fixed paraffin-embedded (FFPE)—but both sequenced under the mRNA-seq protocol. The DI scores confirm that the mRNA transcripts in FFPE samples tend to be highly degraded compared to the paired FF samples (Fig. 3f, l), and degradation patterns are strongly clustered within the same FF or FFPE group (Additional file 2: Figure S1f).

In summary, the DI scores from the DegNorm provide meaningful quantification of gene-level degradation between samples for both cell line and clinical samples under both mRNA-seq and Ribo-Zero-seq protocols. The degradation pattern is gene-specific, and the degradation extent may vary substantially between samples or conditions.

### DegNorm improves accuracy in gene expression analysis

We set out to evaluate how the proposed DegNorm pipeline may improve differential expression analysis by comparing it with other seven normalization methods including UQ [3], TIN [13], RUVr, RUVg [10], trimmed mean of *M* values (TMM) [5], relative log expression (RLE) [24], and total read count (TC) [4]. The RUV methods were designed to remove unwanted variation, but it is unclear whether it is effective for correcting degradation bias. We dropped the RUVg method from the main text for its performance can be very sensitive to the choice of empirical control genes (Additional file 2: Figure S2a-f) or the choice of factor(s) from the factor analysis in the estimation of unwanted variation (Additional file 2: Figure S2 g, h). The TMM, RLE, TC, and UQ methods yielded very similar results in all data we analyzed in this paper (Additional file 2: Figure S3a-f for some examples). For visualization purpose, only the UQ results are presented in the main figures.

**Fig. 4:**
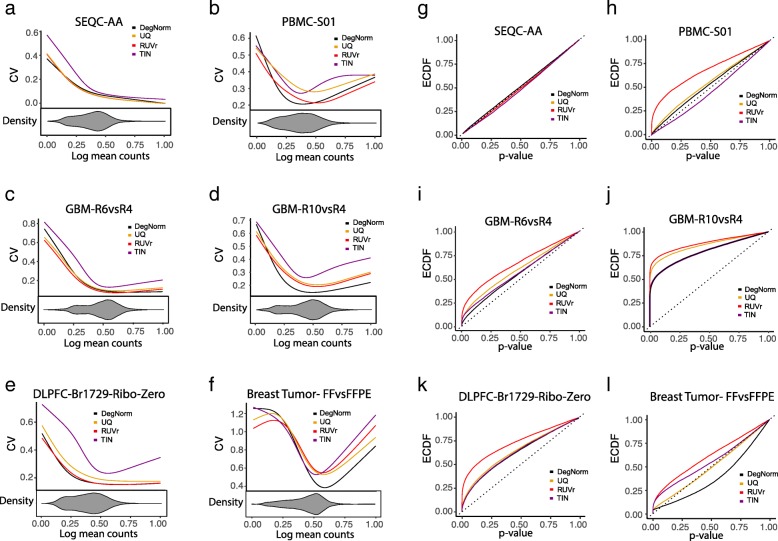
Differential expression (DE) analysis in data sets that had no true differential expression. Results are shown for SEQC-AA, PBMC-S01, GBM R10 + R6, GBM R10 + R4, DLPFC Br1729 Ribo-Zero, and breast tumor data. **a**–**f** Coefficient of variation (CV) vs. mean read counts (in log scale): compared are results from proposed DegNorm pipeline and other methods including upper quartile (UQ), RUVr, and TIN. The mean counts for each data were scaled to 0–1 range by a linear transformation (i.e., (*X*_*i*_ − min_*j*_(*X*_*j*_))/max_*j*_(*X*_*j*_) where *X*_*i*_ is the log of the mean count for gene *i*). The CV curve was generated using the R built-in function smooth.spline. The beanplot under the CV plot shows the density of log of mean read counts from DegNorm (the densities of read counts from other normalization methods are similar and not shown). **g**–**l** Empirical cumulative distribution function (ECDF) of the *p* value from DE analysis. The RUVr results were generated using the RUV-seq package. For TIN method, we followed Wang et al. [13] with details described in the uploaded R Markdown file

We first examine the five data sets that originated from the samples that had no true biological difference between conditions under test (i.e., SEQC-AA, PBMC-S01, GBM, DLPFC, and breast tumor). RNA degradation induces bias and thus may cause extra variance. Severe degradation may even result in a difference of transcript abundance for some genes when they are prepared for RNA-seq. Thus, we investigate how different methods may reduce variance by plotting the coefficient of variation (CV) of normalized read count vs. mean read count in log scale (the logged mean was linearly transformed to 0–1 range, Fig. 4a–f). Overall, the TIN method gives a relatively larger CV than the other three methods. When RNA degradation is a major concern such as in GBM-R10vsR4, and breast tumor FF vs. FFPE comparisons, DegNorm pronouncedly reduced the CV compared to other methods except in the very lower or upper end where the CV was inflated due to outliers (Fig. 4d, f). The RUVr approach applies UQ normalization first and then further removes the additional variation estimated from the factor analysis. It always reduces CV over the UQ method.

We normalized the raw read count by dividing it by 1 - DI score for each gene within each sample. The adjusted read counts (rounded) were input into the edgeR package [25] for DE analysis. When all genes are true nulls, the empirical cumulative distribution function (ECDF) of *p* value tends to be a diagonal line. Thus, an ECDF curve closer to the diagonal line indicates better performance of the normalization method in correcting the degradation bias. For SEQC-AA data with 16 technical replicates, all 4 methods resulted in expected ECDF curves close to the diagonal line (Fig. 4g). For PBMC-S01 and GBM-R6vsR4 comparisons with known modest between-condition difference in mRNA degradation, the ECDF curves from different methods were all well above the diagonal line (except TIN for PBMC data), suggesting that differential degradation probably has caused a difference in gene abundance level when the samples were sequenced (Fig. 4h, i). Both DegNorm and TIN methods brought the ECDF curve down towards the diagonal line compared to UQ and RUVr, indicating that correction for degradation bias helps reduce potential false positives. Nevertheless, the TIN curve in PBMC-S01 data was well below the diagonal line (Fig. 4h), which may indicate a loss of statistical power due to the large variance of the normalized read counts (Fig. 4b). In contrast, the RUVr ECDF curves in both comparisons are significantly higher than that in UQ (regardless that RUVr had lower CV than UQ), suggesting an ineffective correction of degradation bias or even an adverse effect to cause extra false positives. For the GBM-R10vsR4 comparison, ECDF curves are all far above the diagonal line, likely indicating a substantial change of transcript abundance level for many genes due to drastic degradation in R4 samples (Fig. 4j).

The DLPFC and breast tumor FF-FFPE RNA-seq data were both generated from clinical tissue samples. For the DLPFC Br1729 T0+T15 vs. T30+T60 comparison, the DegNorm resulted in a slightly lower *p* value than UQ in Ribo-Zero (Fig. 4k) but much lower than UQ in mRNA-seq (Additional file 2: Figure S4a) data. For the breast tumor data, FFPE samples were shown substantially fragmented and degraded than FF samples (Figs. 1c and 3f). DegNorm resulted in a lower *p* value curve than all other methods (Fig. 4l). We also did cross-protocol DE analysis by comparing the four Br1729 mRNA-seq with four Ribo-Zero samples. All *p* value curves were way above the diagonal line (Additional file 2: Figure S4b), suggesting DE analysis across different sequencing protocols should not be recommended.

**Fig. 5:**
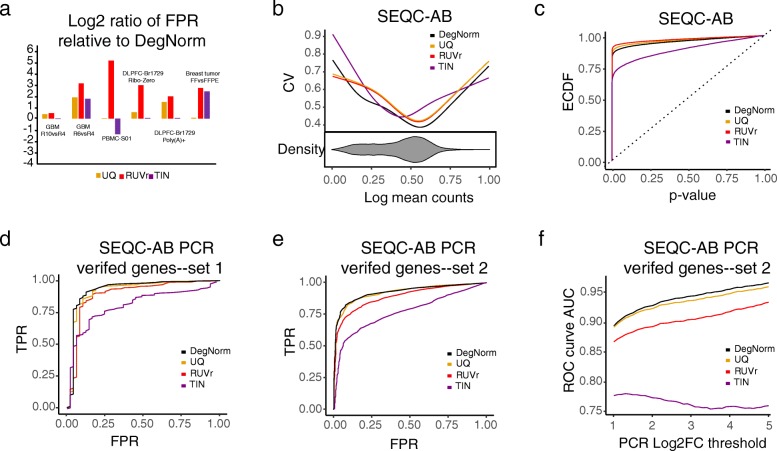
Differential expression analysis results. **a** Log2 ratio of false-positive rate of UQ/RUVr/TIN relative to DegNorm at *q* value = 0.05 criterion for data sets where genes have no differential expression. **b–f** DE results for SEQC-AB data. **b** Coefficient of variation (CV) vs. mean normalized read counts (in log scale). **c** Empirical cumulative distribution function (ECDF) of the *p* value from DE analysis. **d** Receiving operating characteristic curve (ROC) calculated from ~ 500 PCR-verified genes [34]. Genes with absolute log2 fold change value ≥ 2 were defined as true positives, and absolute log2 fold change value ≤ 0.1 was defined as true negatives. Genes with log2 fold change in between were disregarded. **e** ROC calculated based on ~ 17,304 PCR-verified genes published in a separate study [27] for the same SEQC AB samples. The same threshold values of log2 fold change as in **c** were used in defining the positives and negatives. **f** The area under the ROC curve (AUC) statistic as a function of the threshold value of absolute log2 fold change used to define the true positives based on the PCR data from **e**

The *p* value ECDF curve provides a global picture of a false-positive rate at different type-I error rate thresholds when all null hypotheses are true. In practice, as the ground truth is unknown, one typically claims the DE by controlling the false discovery rate (FDR) to correct multiple comparison errors. Thus, we further compared the false-positive rate of different methods by controlling the nominal FDR under the criterion of *q* value ≤ 0.05 using q-value package [31–33]. For SEQC-AA data with a little degradation difference between samples, all four methods resulted in very few claimed positives (Additional file 3: Table S1), consistent with the close-to-diagonal-line nature of *p* value ECDF curves in Fig. 4g. For the rest data with differential degradation, all methods yielded a substantial number of false positives. For comparison, we plotted the ratio of the false-positive rate of UQ, RUVr, and TIN over DegNorm in log2 scale (Fig. 5a). The UQ and RUVr methods consistently yielded more false positives than DegNorm, by a factor ranging from 1 to 3.7 and 1.4–36.8, respectively. The TIN method reduced the false-positive rate over DegNorm in the PBMC-S01 with a factor of 1.37. Nevertheless, we will show below that this relatively lower false-positive rate from the TIN method is an indication of undermined power due to the excessively inflated variance (Fig. 4a–f).

The SEQC-AB data presents an atypical example containing an unusually large number of differentially expressed genes [27, 34]. DegNorm produced an overall lower CV than UQ, RUVr, and TIN methods (Fig. 5b). The DegNorm ECDF lies below those of UQ and RUVr methods while above TIN (Fig. 5c). With the presence of truly differentially expressed genes, the lower ECDF can be interpreted as a tendency to result in fewer false positives (good) or more false negatives (bad, less power) or a mix in the DE analysis. To investigate this, we utilized 2 sets of PCR-verified genes, 1 from the original MAQC study of 843 genes [34] and the other from SEQC study of 20,801 genes [27], as the ground truth to construct receiver operating characteristic curves (ROC) (Fig. 5d, e). Similar to Rissio et al. [10], we defined a gene as a positive if the absolute value of log2 fold change ≥ 2, a negative if ≤ 0.1, and undefined otherwise. For both sets, the ROC curves suggest that DegNorm achieved better true-positive rate (sensitivity) than UQ, RUVr, and TIN while controlling the false-positive rate (1-specificity). For example, at FPR = 0.05 (specificity = 0.95), the larger PCR set suggested a 78.1% true-positive rate for DegNorm in comparison with 73.7%, 67.3%, and 48.3% for UQ, RUVr, and TIN methods, respectively. When we varied the of log2 fold change threshold value from 1 to 5 to define the positives, the area under the ROC curve (AUC) from all methods (except TIN) increased as expected, while the AUC from DegNorm remained the largest and the gap between DegNorm and other methods enlarge (Fig. 5f). As the true-negative set was fixed in this experiment, the true-positive rate drove the change of AUC as the threshold value increases. This suggests DegNorm improves the power to identify highly differentially expressed genes over other normalization methods while controlling the false-positive rate. Therefore, we conclude that the lower ECDF curve from DegNorm method (Fig. 5c) manifests a good tendency to reduce false-positive rate or increase specificity without sacrifice of statistical power or sensitivity.

The RUVr and TIN methods both showed pronouncedly lower power than DegNorm and UQ, but due to different reasons (Fig. 5d–f). The RUVr method is guaranteed to reduce more variation based on the UQ-normalized data. Nevertheless, the removed variation may contain true biological difference if it is confounded with unwanted variation, which will result in more false negatives. On the other hand, the large variance incurred by TIN method appears to severely undermine the power in the DE analysis (Fig. 5b).

**Fig. 6:**
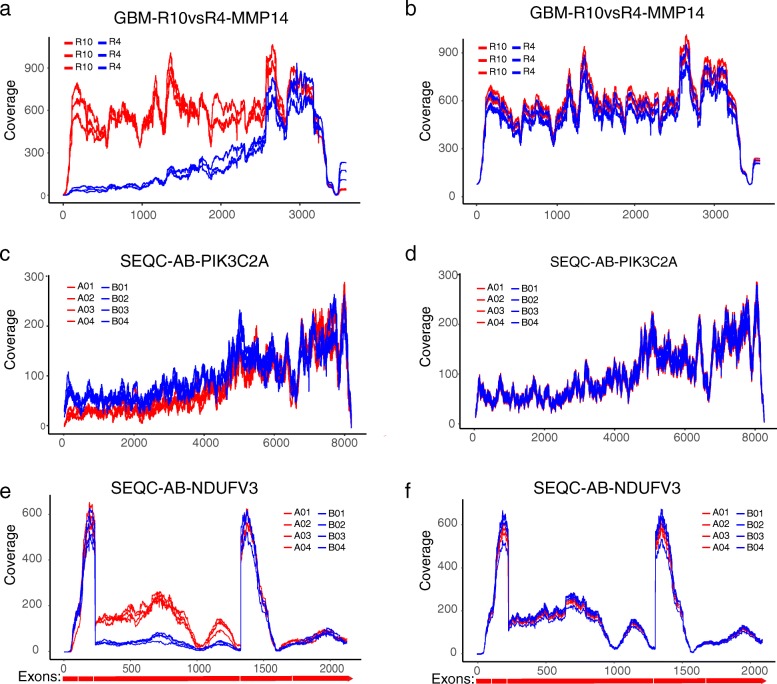
Examples illustrating that DegNorm improves accuracy in RNA-seq DE analysis. **a**, **b** Coverage curves under UQ and DegNorm normalization methods side-by-side for gene MMP14 in GBM R10vsR4 comparison. Same coverage plots for PIK3C2A (**c**, **d**) and NDUFV3 (**e**, **f**) genes, respectively, in the SEQC-AB comparison. All three genes changed from positive to negative after degradation normalization in DE analysis using edgeR. In (**e**, **f**), annotated exon boundaries are plotted to show possible alternative splicing in the exon from 223 to 1327 nt

We present three examples that exemplify how DegNorm may improve the accuracy in the DE analysis. We used local false discovery rate (lfdr) from q-value package [31–33] to quantify the significance of DE analysis. Compared to *q* value, which quantifies the average FDR for all genes with smaller *p* value than the given one, lfdr is more appropriate to quantify the false discovery rate associated with any individual *p* value. The MMP14 gene in the GBM-R10vsR4 comparison displayed a clear degradation in the 5′ end in R4 samples (Fig. 6a) and tested positive using UQ method (*p* value = 1.31e−8, lfdr = 1.1e−8). DegNorm compensated for the degraded portion of R4 samples and returned negative test result (*p* value = 0.91, lfdr = 0.32, Fig. 6b). The second example is the PIK3C2A gene from SEQC-AB comparison, a PCR-verified negative with log2 fold change = 0.06. It tested positive under UQ (*p* value = 0.005, lfdr = 0.02) (Fig. 6c) while negative under DegNorm with degradation correction in the 5′ end of A samples (*p* value = 0.77, lfdr = 0.97, Fig. 6d). The third example is the NDUFV3 gene from the SEQC-AB data, showing nearly depleted coverage in the entire third exon region from ~ 223 to 1327 nt for B samples likely due to alternative splicing. It tested positive under UQ (*p* value = 1.94e−37, lfdr = 3.39e−09, Fig. 6e). DegNorm returned a negative result (*p* value = 0.808, lfdr = 0.942, Fig. 6f), consistent with negative PCR verification (log2 fold change = − 0.114).

### GBM AMPK knockout data

**Fig. 7:**
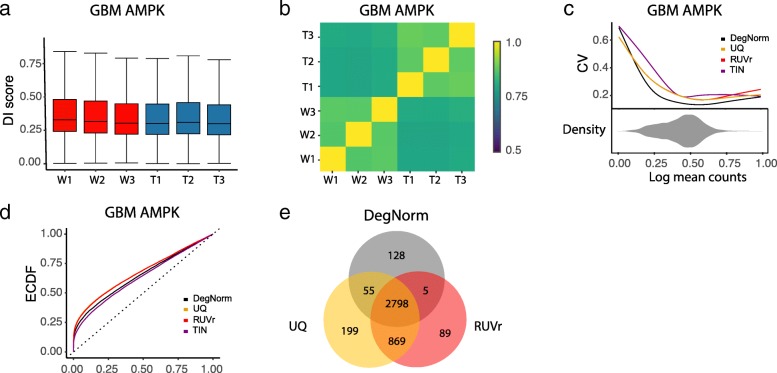
DE analysis in GBM AMPK knockout data. **a** Box plot of estimated DI scores where condition W and T stand for control and knockout samples, respectively. **b** Correlation plot of DI scores shows within condition replicates had a higher correlation of degradation pattern than between condition samples. **c** Coefficient of variation (CV) vs. mean normalized read counts (in log scale). The bean pot under the CV plot shows the density of log of mean read counts from DegNorm. **d** Empirical cumulative distribution function (ECDF) of the *p* value from DE analysis. **e** Venn-diagram shows the number of claimed differentially expressed genes and pairwise overlap by DegNorm, UQ, and RUVr methods at *q* value threshold = 0.05

GBM AMPK data presents a typical case in a clinical study where RNA-seq is used to survey a transcriptome for differential expression between control and treatment samples. DegNorm uncovered higher degradation in the control than the AMPK knockout samples (Fig. 7a) and heterogeneity in degradation pattern between the two conditions (Fig. 7b). DegNorm also resulted in a lower CV curve and a lower ECDF of *p* value than UQ and RUVr methods (Fig. 7c, d). We claimed differential expression under *q* value ≤ 0.05 and compared the test-positive sets between DegNorm, UQ, and RUVr (Fig. 7e). There were 2798 positive genes shared by all 3 methods, while UQ and RUVr produced 935 and 775 more positives than DegNorm, respectively. We suspect degradation may cause an excess of false positives (as suggested by similar plots in Fig. 6 for SEQC-AB data); nevertheless, without a large set of PCR-verified genes, it is impossible to rigorously assess the sensitivity and specificity. Chhipa et al. [30] analyzed the RNA-seq data and concluded that the bioenergetics of cellular metabolism was the most significantly downregulated pathway in AMPK-depleted samples. They applied RT-qPCR and verified a small set of downregulated genes using independent GBM cell lines from 3 other patients. Among the 12 genes verified as downregulated, including HIF1a, LDHA, SLC2A1, HK1, GPI, ALDOA, TPI1, PFKM, ENO1, GABPA, TFAM, and COX20, DegNorm, UQ, and RUVr all successfully identified 8 except HK1, PFKM, GABPA, and COX20, whereas TIN method missed 2 additional genes, GPI and ALDOA (Additional file 4: Table S2). The discrepancy between the RNA-seq set and PCR results could be due to cell line-specific variation, lack of power due to small sample size or sample quality (personal communication with Dr. Dasgupta). Clearly, such a small-scale verification is insufficient to conclude about the sensitivity, neither can we evaluate the specificity without verification of the negatives. The discrepancy between DegNorm and other methods does provide alerts to users of possible false positives caused by degradation when interpreting the results of such studies.

### Simulation study

To further systematically investigate the performance of DegNorm, we conducted a simulation study in a two-condition comparison: 4 control (A) vs. 4 treatment (B) samples in 4 different degradation settings. Each sample of 20,000 genes was simulated with a random sequencing depth of 40–60 million reads, 5% of which were chosen to be upregulated and another 5% for downregulated in expression. In the first setting, all genes had no degradation whereas in the rest 3 settings, 80% of the genes were randomly selected for degradation. In the second setting, for a gene selected for degradation, either 3, 4, or 5 samples out of the 8 were randomly chosen for degradation, whereas in the third, either all 4 control samples or 4 treatment samples were randomly chosen for degradation. In both second and third settings, the degradation extent for each degraded gene was random but following the same distribution. In the fourth setting, for each gene selected to degrade, 2 control samples were randomly selected for degradation with the same expected severity, while all treatment samples had a sample-specific systematic difference in expected severity. The simulation details are described in the “Methods” section and Additional file 1.

**Fig. 8:**
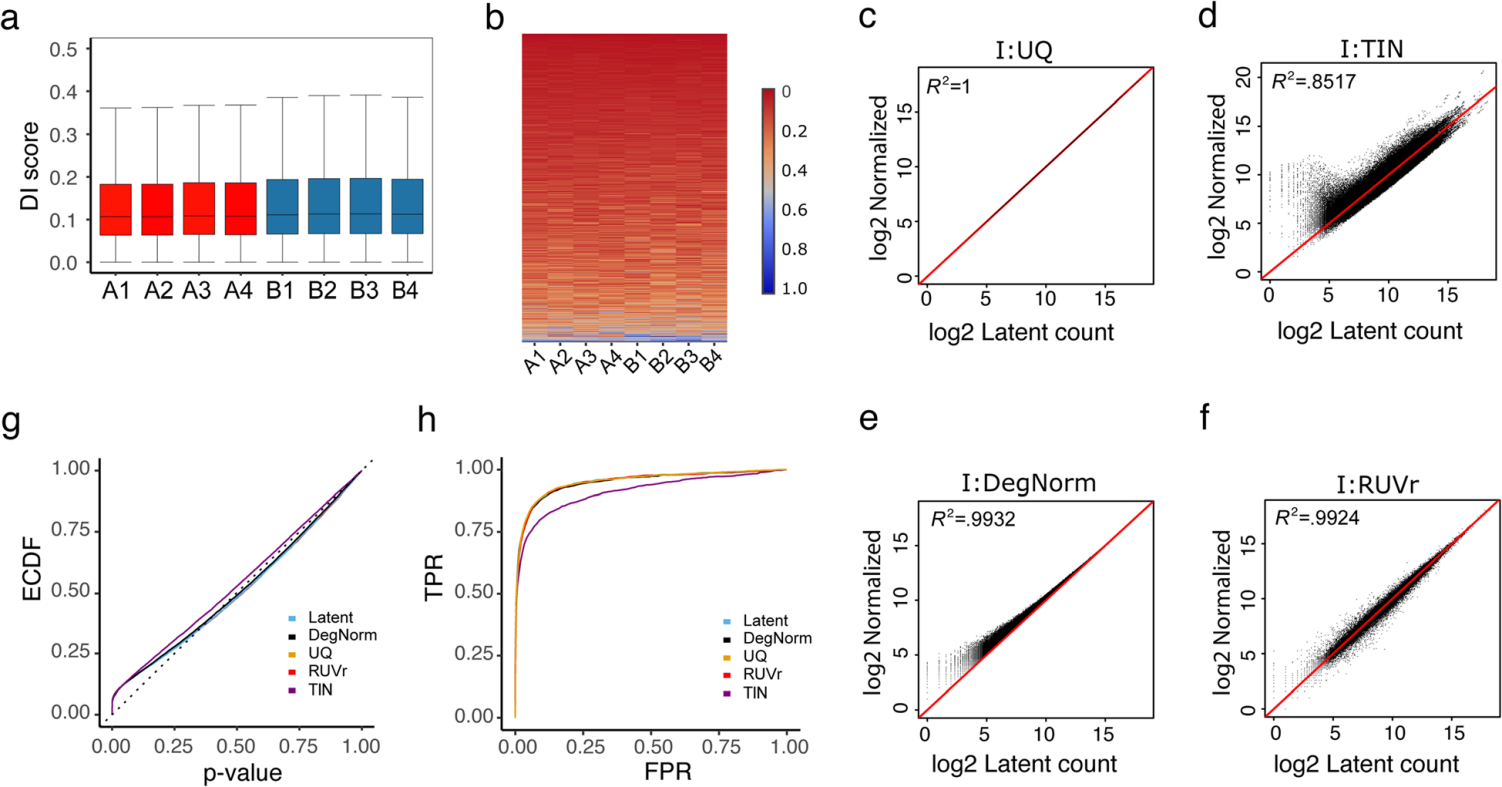
Results for simulation I. **a** Box plot of estimated DI scores where conditions A and B stand for control and treatment samples, respectively. **b** Heatmap of DI scores of genes sorted in the ascending order of the average DI score of condition A (control), showing no between-condition clustered pattern. **c**–**f** Scatter plot of normalized read count vs. latent read count in log2 scale with diagonal line imposed for simulation I. Compared are results from proposed DegNorm pipeline and other methods including upper quartile (UQ), RUVr, and TIN. **g**, **h** ECDF plots of *p* value and ROC curves for simulation I under different normalization methods. All curves except TIN were well overlapping with each other, suggesting close performance in DE analysis

Simulation I presents a scenario where samples have no degradation bias or any other bias but only between-sample variation in sequencing depth. In the following, we shall refer the latent count as the true read count for a gene before degradation is imposed. DegNorm is data driven and always returns non-negative DI scores by design. The estimated DI scores have a median ~ 0.11 in all samples (Fig. 8a), demonstrating the absence of between-condition heterogeneity (Fig. 8b). To investigate how the positive bias in DI scores may impact DE analysis, we first plotted the normalized vs. latent read count from different methods (Fig. 8c–f) (the latent read count in simulation I is just the raw read count). Unsurprisingly, the UQ method perfectly normalizes the sequencing depth (Fig. 8c), while all other three methods caused bias or extra variance to different extents (Fig. 8d–f). The positive bias from DegNorm is more pronounced when read counts are low such that read coverage curve cannot be well estimated (Fig. 8e). As for any gene, all samples are subject to this over-estimation bias; this bias may partially cancel off in DE analysis. As a result, the *p* value ECDF and ROC curves of DegNorm almost perfectly overlap with the latent and UQ curves (Figs. 8g, h). At FPR = 0.05, DegNorm, RUVr, and TIN had sensitivity decay of 0.6%, 1.1%, and 8.6%, respectively, compared to using the latent counts or UQ method (Additional file 5: Table S3).

**Fig. 9:**
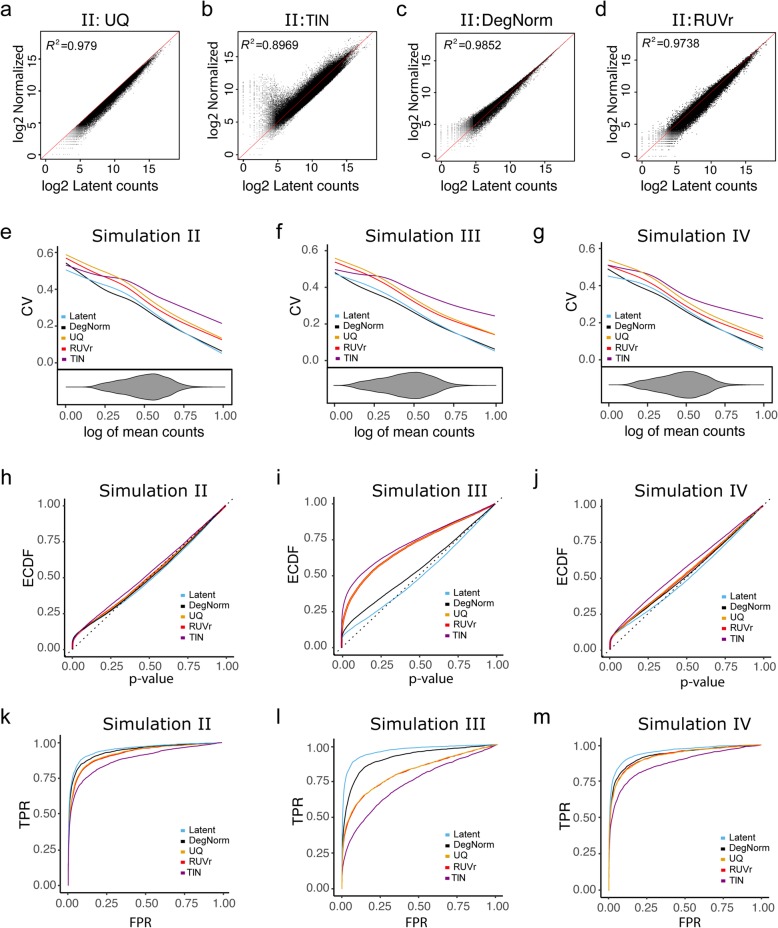
Comparison of different normalization methods in simulations II, III, and IV. **a**–**d** Scatter plot of normalized read count vs. latent read count in log2 scale with diagonal line imposed from DegNorm, UQ, RUVg, RUVr, and TIN methods for simulation II. **e**–**g** Coefficient of variation (CV) vs. normalized mean read count (in log scale) for 18,000 true-negative genes (out of a total of 20,000 genes). The bean pot under the CV plot shows the density of log of mean read counts from DegNorm. **h**–**j** Empirical CDF of *p* value from edgeR DE analysis. The latent curve corresponds to the results using the true read counts before the degradation was imposed. **k**–**m** Receiver operating characteristic curves of DE analysis

When degradation is an issue as in simulations II–IV, the estimated DI scores provide an informative characterization of the overall degradation severity of each sample (Additional file 2: Figure S5a-f) as well as the similarity of gene-specific degradation pattern between samples (Additional file 2: Figure S5 g-l). DegNorm demonstrates consistently better correction of degradation bias than the UQ, TIN, and RUVr methods, as evidenced by higher regression coefficient of determination (*R*^2^), and a nearly symmetric distribution of data points around the diagonal line in the scatter plot of normalized vs. latent read counts (Fig. 9a–d, Additional file 2: Figure S6a-h). The UQ method does not correct for any degradation bias but instead only normalizing sequencing depth (Fig. 9a, Additional file 2: Figure S6a, e). Similar extreme variance issue is also observed in the TIN method in simulations II–IV (Fig. 9b, Additional file 2: Figure S6b, f).

To gain more insights into the CV plots, we singled out the 18,000 truly non-differentially expressed genes from simulations II–IV and plotted the CV against the mean of normalized count (Fig. 9e–g). Without the confounding effect of biological difference, the CV of these genes tends to be inflated due to degradation-caused loss of read count. Thus, the CV is an informative measure to assess the effectiveness of degradation normalization. Indeed, the UQ and RUVr curves are both well above the latent curve in all simulations, suggesting degradation bias was not or inadequately corrected. Similar to what we observed in the real data, the TIN CV curve dominates other methods in each setting, echoing the excess of variance observed in the scatter plot (Fig. 9b, Additional file 2: Figure S6b, f). In contrast, the DegNorm curves were the closest to the respective latent curves but with a slight underestimation of CV in the lower half.

**Fig. 10:**
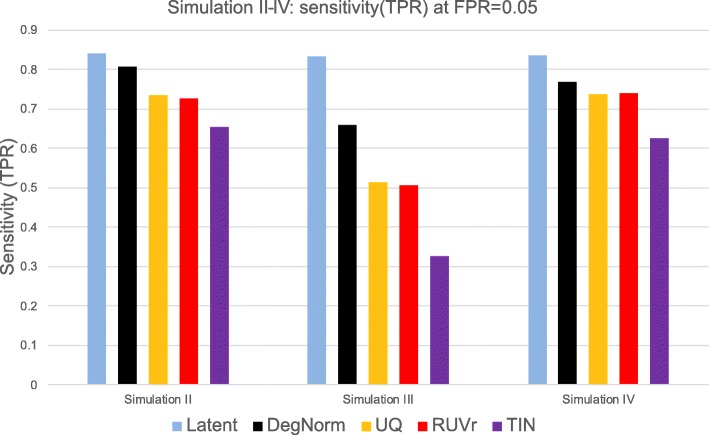
Bar chart of sensitivity (TPR) of different methods in simulations II, III, and IV. True-positive rate (sensitivity) is compared at false-positive rate threshold = 0.05 for each method in each simulation

DegNorm improves the accuracy in DE analyses. In both ECDF (Fig. 9h–j) and ROC plots (Fig. 8k–m), the DegNorm curve was the closest to the latent one, demonstrating improvement over other normalization methods to different extents. We tabulated the sensitivity of each method at FPR = 0.05 (Additional file 5: Table S3) and plotted it in Fig. 10. In settings II and III, where degradation was randomly chosen among samples (II) or conditions (III), the UQ and RUVr methods were both ineffective to correct this non-systematic but gene-specific bias (Figs. 9k, l and 10). In particular, in simulation III as degradation was applied to one condition of random choice for a given gene, the degradation bias was completely confounded with the covariate of interest and cannot be removed by UQ or RUVr method. Consequently, many false positives were called due to degradation bias (Fig. 9l). At FPR = 0.05 threshold, DegNorm improved the sensitivity by a factor of 1.28, 1.30, and 2.01 compared to UQ, RUVr, and TIN methods, respectively (Fig. 10, Additional file 5: Table S3). In contrast, the treatment samples in setting IV had a systematic difference in average degradation (Additional file 2: Figure S5c), both UQ and RUVr performed reasonably well (Figs. 9m and 10). In all four degradation settings, the TIN method showed inferior power to detect true DE genes.

## Discussion and conclusions

In this paper, we showed that RNA degradation pattern and severity are not only sample specific, but also gene-specific, and thus commonly used global normalization methods that impose a sample-specific constant adjustment to all genes within the same sample are ineffective to correct for this bias. The RUVr approach is guaranteed to reduce variation, while they failed to show pronounced improvement over the UQ method in the DE analysis in all data sets considered in this study (even got worse in all data under consideration). One complexity is that the true biological difference is often confounded with degradation (e.g., SEQC-AB data) and other unwanted variation. The factor analysis cannot well separate the unwanted from the wanted variation, and it may even remove the true biological difference of interest. This confounding issue was illustrated in the SEQC-AB data by the high within-condition and low between-condition correlation of DI scores (Additional file 2: Figure S1b). In particular, the RUVg method was sensitive to the selection of empirical control genes or the factor(s) used to estimate the unwanted variation (Additional file 2: Figure S2a-j). More objective criteria in this regard need to be developed for the RUVg method.

Although motivated by mRNA degradation, we defined the degradation in this paper in a generalized and relative sense. The quantified DI scores may reflect confounding effects from mRNA degradation, alternative splicing, and other factors. Risso et al. [10] showed that in the SEQC-AB data, samples were clustered due to the difference of sample preparation, experiment protocol, sequencing run batches and flow cell, etc. Such factors could impact the RNA samples by changing the read coverage curves. Thus, normalizing heterogeneity in coverage curves may help reduce bias due to such factors. Indeed, in all five benchmark data sets considered in this paper, DegNorm performed consistently better compared to other methods regardless of whether mRNA degradation was a known concerning issue. Unlike mRIN and TIN measures where degradation was defined as the deviation from hypothesized uniform coverage curve, the DI score from DegNorm is defined with reference to an adaptively estimated latent coverage curve that minimizes the distance to the observed coverage curves. If a gene has 50% degradation but having consistent coverage curves across samples, the estimated DI scores will all be nearly 0. In this case, the read count in each sample can still accurately reflect the relative abundance between samples, and degradation correction is unnecessary. From this perspective, the DI score is defined in a relative sense. Normalizing the read counts for degradation bias using DI scores is hoped to minimize the extrapolation needed, thus avoids an excess of variance. The advantage of this strategy was exemplified in all real and simulated data sets in contrast to the TIN method.

There are a few limitations of the DegNorm method. First, like any other normalization method, DegNorm is a post hoc approach that is designed to alleviate the issues due to degradation heterogeneity between samples/genes and thus improve the accuracy of DE analysis. It cannot completely remove the bias for every single gene. In particular, cautions must be taken when testing DE for samples that have a dramatic difference in degradation (e.g., GBM R10 vs. R4), as it may change the true abundance level of transcripts of interest and lead to the excess of false positive or false negatives. High-quality RNA samples are always desirable in RNA-seq. Furthermore, cross-platform DE analysis is not recommendable. Second, the core component of DegNorm is a matrix factorization over-approximation algorithm aiming to correct for the degradation bias that commonly exists in RNA samples, even for the high-quality SEQC data. DegNorm tends to result in a pronounced over-estimation bias in DI scores for genes with low read counts regardless of whether degradation is present (simulations II–IV) or absent (simulation I). We showed in simulation I that this bias is not a big concern in DE analysis as it tends to be homogeneous across all samples for all non-degraded genes. Third, DegNorm has only been tested in this paper on the bulk RNA-seq data generated from a cell line or clinical samples (FF or FFPE) under mRNA-seq or Ribo-Zero-seq protocol. The effectiveness of DegNorm for RNA-seq data from 3′ end sequencing (3seq) or other variants needs to be further investigated in the future. Lastly, DegNorm is computing intensive due to parsing read alignment results, calculation of read coverage curves, and repeated non-negative matrix factorization of large matrices. We have implemented DegNorm in a Python package available at https://nustatbioinfo.github.io/DegNorm/. Currently, it took about 9 h to run the entire pipeline on the FF vs. FFPE comparison (6 samples) on a 22-core node on the Linux cluster. We have implemented an MPI release through parallel computing that can reduce the time by a factor of *n* where *n* is the number of nodes used.

In summary, we conclude DegNorm provides a pipeline for informative quantification of gene-/sample-specific transcript degradation pattern and for effective correction of degradation bias in RNA-seq. We intend for DegNorm to serve as a general normalization method to improve the accuracy in the gene expression differentiation analysis.

## Methods

### DegNorm algorithm

Suppose we have *p* samples and *n* genes, *X*_*ij*_ is the read count for gene *i* in sample *j*. For simplicity of notation, we first illustrate the proposed method by focusing on one gene. Let *f*_*ij*_(*x*), *x* = 1, … , *L*_*i*_; *i* = 1, … , *n*; *j* = 1, … , *p* be the read coverage score for transcript *i* of length *L*_*i*_ from sample *j*. When different isoforms are present, the *L*_*i*_ positions represent the assembly of all expressed exons in the sequential order. We assume there is an *envelope* function *e*_*i*_(*x*) that defines the ideal shape of read coverage curve for gene *i* if no degradation exists. The actual ideal coverage curve for the given gene in the *j*th sample is *k*_*ij*_*e*_*i*_(*x*), where *k*_*ij*_ denotes the confounded effect of sequencing depth and relative abundance of gene *i* in sample *j*.

Degradation causes *f*_*ij*_(*x*) to deviate downward from *k*_*ij*_*e*_*i*_(*x*) in degraded region(s). Clearly, sampling error can cause random fluctuation of *f*_*ij*_(*x*) from the ideal curve *k*_*ij*_*e*_*i*_(*x*) (Fig. 2a, b). We assume the random error is negligible compared to the major bias arising from degradation. Thus we require *k*_*ij*_*e*_*i*_(*x*) ≥ *f*_*ij*_(*x*) for all *j* and *x*. The difference between *k*_*ij*_*e*_*i*_(*x*) and *f*_*ij*_(*x*) provides an estimate of degraded portion of read count. We propose a method that allows to estimate *k*_*ij*_ and *e*_*i*_(*x*) to quantify the degradation extent of each gene within each sample while simultaneously controlling the sequencing depth.

#### Estimating degradation via non-negative matrix over-approximation

Let **f**_*ij*_ = (*f*_*ij*_(1),  … , *f*_*ij*_(*L*_*i*_))^*T*^, *j* = 1, … , *p* and **F**_***i***_ = (**f**_*i*1_,  … , **f**_*ip*_)^*T*^. Let **K**_***i***_ = (*k*_*i*1_,  … , *k*_*ip*_)^*T*^, **E**_***i***_ = (*e*_*i*_(1),  … , *e*_*i*_(*L*_*i*_))^*T*^. We propose to estimate **K**_***i***_ and **E**_***i***_ by minimizing the following quadratic loss function subject to some constraint:

$$ Q\left(\ {\mathbf{K}}_{\boldsymbol{i}},{\mathbf{E}}_{\boldsymbol{i}}\right)=\sum \limits_{x=1}^{L_i}\sum \limits_{j=1}^p{\left[{k}_{ij}{e}_i(x)-{f}_{ij}(x)\right]}^2\ \mathrm{s}.\mathrm{t}.{k}_{ij}{e}_i(x)-{f}_{ij}(x)\ge 0,{k}_{ij},{e}_i(x)>0,\forall j,\forall x. $$

We can configure this problem into a non-negative matrix factorization problem [35, 36] as follows:

$$ \underset{{\mathbf{K}}_{\boldsymbol{i}},{\mathbf{E}}_{\boldsymbol{i}}}{\min }{\left\Vert {\mathbf{K}}_{\boldsymbol{i}}{{\mathbf{E}}_{\boldsymbol{i}}}^T-{\mathbf{F}}_{\boldsymbol{i}}\kern0.2em \right\Vert}^2\kern0.2em \mathrm{s}.\mathrm{t}.{\mathbf{F}}_{\boldsymbol{i}}\le {\mathbf{K}}_{\boldsymbol{i}}{{\mathbf{E}}_{\boldsymbol{i}}}^T,{\mathbf{K}}_{\boldsymbol{i}}\ge \mathbf{0},{\mathbf{E}}_{\boldsymbol{i}}\ge \mathbf{0}, $$

where ‖**∙**‖^2^ stands for the element-wise quadratic norm (i.e., sum of squared elements), and ≤ and ≥ for element-wise logical comparison. We call this a rank-one non-negative matrix factorization over-approximation (NMF-OA) problem as **K**_***i***_ and **E**_***i***_ both have rank 1 and **K**_***i***_**E**_***i***_^***T***^ ≥ **F**_***i***_. The proposed iterative algorithm is described in details in the Additional file 1.

#### Refinement of NMF-OA algorithm

The NMF-OA optimization algorithm provides an approximate solution to this problem. However, in the RNA-seq data, the performance of the solution can be affected by two confounding factors, the degradation extent and the sequencing depth. In our quadratic objective function *Q*(**K**_***i***_, **E**_***i***_), an **f**_*ij*_ of larger magnitude tends to have more influence on the estimation of envelope function. A dominant scale in **f**_*ij*_ may force the algorithm to fit an envelope function that resembles **f**_*ij*_ to minimize the loss. Thus, a good scale normalizing factor for sequencing depth is important to yield a good estimate of the envelope function *e*_*i*_(*x*). With gene-specific and sample-specific degradation, the total number of reads may not provide a reliable measure of sequencing depth.

Second, given **F**_***i***_ that is appropriately normalized for sequencing depth, the scale factor **K**_***i***_ should reflect the relative abundance of the gene in the non-degraded region of each sample (to be referred to as the baseline region below) (Fig. 2a). The non-degraded region must preserve a similar shape in gene’s coverage curves from different samples. If one can first estimate **K**_***i***_ from the identified baseline region, then NMF-OA algorithm will lead to a better estimate of the envelope function **E**_***i***_, particularly in the situation when the degradation extent is severe as the GBM data. We account for these two considerations by proposing an iterative degradation normalization pipeline (DegNorm) as follows:

1. Sequencing depth adjustment: given the current estimate of (**K**_***i***_***,*E**_***i***_) for *i* = 1, … , *n*, define the degradation index (DI) score as:

$$ {\rho}_{ij}=1-\frac{\sum_{x=1}^{L_i}{f}_{ij}(x)}{\sum_{x=1}^{L_i}{k}_{ij}{e}_i(x)}. $$

Graphically, *ρ*_*ij*_ stands for the fraction of the total area under the curve *k*_*ij*_*e*_*i*_(*x*) but above *f*_*ij*_(*x*) (Fig. 2e). The DI score is used to calculate an adjusted read count by extrapolation:

$$ {\overset{\sim }{X}}_{ij}=\frac{X_{ij}}{1-{\rho}_{ij}}. $$

Next, we calculate the sequencing depth scaling factor using the degradation-corrected total number of read count:

$$ {s}_j=\frac{\sum_{i=1}^n{\overset{\sim }{X}}_{ij}}{{\mathrm{Median}}_{\mathrm{j}}\left\{{\sum}_{i=1}^n{\overset{\sim }{X}}_{ij}\right\}}. $$

In this paper, the results presented were all based on this scale normalization. Alternatively, we can use other normalization methods like TMM or UQ to calculate the normalizing constant *s*_*j*_ based on the degradation-adjusted read count ${\overset{\sim }{X}}_{ij}$ if the presence of extreme outliers is a concern.

2. Degradation estimation: given the estimated sequencing depth *s*_*j*_ from step 1, adjust the coverage curves as follows:

$$ {\mathbf{f}}_{ij}\leftarrow \frac{{\mathbf{f}}_{ij}}{s_j}. $$

- Let **F**_***i***_ = (**f**_*i*1_,  … , **f**_*ip*_)^*T*^. Run NMF-OA for each gene on updated coverage curves **F**_***i***_ and obtain the estimate of (**K**_***i***_***,*E**_***i***_).
- Divide each gene into 20 bins. Define the residual matrix as **R**_**i**_ = **K**_***i***_**E**_***i***_^***T***^ − **F**_***i***_ . We identify a subset of bins on which **F**_***i***_ preserves the most similar shape as the envelope function **E**_***i***_ across all samples by progressively dropping bins that have the largest sum of squares of normalized residuals (**R**_**i**_/**F**_***i***_) and repeatedly applying NMF-OA to the remaining bins. This step stops if the maximum DI score obtained from the remaining bins is ≤ 0.1 or if 80% bins have been dropped (see details in Additional file 1). The remaining transcript regions on selected bins are regarded as the baseline. Denote the read coverage curve on baseline region as ${\mathbf{F}}_{\boldsymbol{i}}^{\ast}$.
- Run NMF-OA on ${\mathbf{F}}_{\boldsymbol{i}}^{\ast}$. The resulting ${\mathbf{K}}_{\boldsymbol{i}}^{\ast}$ is a refined estimate of **K**_***i***_, given which the envelope function can be obtained as:

$$ {e}_i(x)={\max}_j\left\{\frac{f_{ij}(x)}{k_{ij}^{\ast }}\right\},x=1,\dots, {L}_i. $$

3. Steps 1 and 2 are repeated until the algorithm converges.

The DE analysis using edgeR was carried out based on the degradation normalized read count ${\overset{\sim }{X}}_{ij}$ (rounded) at convergence with upper quartile (UQ) normalization for sequencing depth.

### Simulations

We first simulated the latent read count for each gene within each sample. For a given gene without DE, the latent counts (without degradation) in both control and treatment samples were randomly simulated from the negative binomial distribution with the same mean and same dispersion parameters that were randomly chosen from the fitted values of SEQC-AB data. For a gene with DE, the latent counts for the control samples were first simulated as above, and those for the treatment samples were simulated from the negative binomial with mean parameter increased/decreased by a factor of (1.5 + *γ*) for up- or downregulated genes, respectively, where *γ* was simulated randomly from an exponential distribution with mean = 1. In the second step, we simulated the degradation given the latent read count (simulations II–IV). For a given gene, we first chose a Gaussian mixture distribution that covers the entire range of total transcript to model the read start position distribution. For simplicity, we only consider 5′ end degradation. Each read from a gene that was pre-selected for degradation degraded (or was disregarded) with the probability that depended on the start position of the read, defined by a cumulative distribution function (CDF) of a lognormal distribution. The degradation pattern and extent can be tuned by varying the parameters in the lognormal distribution (see more details in Additional file 1).

## Additional files

Additional file 1: Supplementary methods. (DOCX 109 kb)

Additional file 2: Supplementary figures and figure legends. (PDF 69270 kb)

Additional file 3: **Table S1.** This table summarizes the number of false positives claimed in differential expression analysis by edgeR from different normalization methods at *q* value threshold = 0.05 for data sets including SEQC-AA, GBM RIN10 vs. RIN4, GBM RIN6 vs. RIN4, PBMC-S01, DLPFC Br1729 Ribo-zero, DLPFC Br1729 poly(A)+, and breast tumor FF vs. (XLSX 9 kb)

Additional file 4: **Table S2.** This table compares the *p* values and *q* values of differential expression analysis by edgeR from different normalization methods for 12 genes which were PCR-verified positives in the AMPK data. (XLSX 12 kb)

Additional file 5: **Table S3.** This table compares the sensitivity (true-positive rate) achieved by different normalization methods in the differential expression analysis at FPR (false-positive rate) threshold = 0.05 for simulations I–IV. (XLSX 8 kb)

Additional file 6: Review history. (DOCX 53 kb)
