# Supplementary material for: DegNorm: normalization of generalized transcript degradation improves accuracy in RNA-seq analysis
Source: Genome Biol. 2019 Apr 16;20:75. doi: 10.1186/s13059-019-1682-7 (PMC6466807; doi:10.1186/s13059-019-1682-7)
Supplement: Supplementary file 1 — Supplementary methods. (DOCX 109 kb) [file 13059_2019_1682_MOESM1_ESM.docx]

## Supplementary Methods

## Nonnegative matrix factorization --- over approximation

Let $\text{f}_{ij}={(f_{ij}\left( 1 \right),\ldots,f_{ij}\left( L_{i} \right))}^{T}, j=1,\ldots,p$ and $\text{F}_{\boldsymbol{i}}\text{ }\text{=}{(\text{f}_{i1},\ldots,\text{f}_{ip})}^{T}$ be the observed coverage curves for gene $i$. Let $\mathbf{K}_{\boldsymbol{i}}={(k_{i1},\ldots,k_{ip})}^{T}$, $\mathbf{E}_{\boldsymbol{i}}={(e_{i}\left( 1 \right),\ldots,e_{i}\left( L_{i} \right))}^{T}$. We propose to estimate $\mathbf{K}_{\boldsymbol{i}}$ and $\mathbf{E}_{\boldsymbol{i}}$ by minimizing the following quadratic loss function subject to some constraint:

$$Q\left( \mathbf{K}_{\boldsymbol{i}}\text{, }\mathbf{E}_{\boldsymbol{i}} \right)=\sum_{x=1}^{L_{i}} \sum_{j=1}^{p} \left[ k_{ij}e_{i}\left( x \right)-f_{ij}\left( x \right) \right]^{2} \text{s.t}. k_{ij}e_{i}\left( x \right)-f_{ij}\left( x \right)\geq0,k_{ij},e_{i}\left( x \right)>0, \forall j, \forall x.$$

We can configure this problem into a non-negative matrix factorization problem as:

$$\min_{\mathbf{K}_{\boldsymbol{i}}\text{,}\mathbf{E}_{\boldsymbol{i}}} {\text{||}\mathbf{K}_{\boldsymbol{i}}{\mathbf{E}_{\boldsymbol{i}}}^{\boldsymbol{T}}-\text{F}_{\boldsymbol{i}}\text{ }||}^{2} s.t. \text{F}_{\boldsymbol{i}}\text{ }\text{≤} \mathbf{K}_{\boldsymbol{i}}{\mathbf{E}_{\boldsymbol{i}}}^{\boldsymbol{T}}, \mathbf{K}_{\boldsymbol{i}}\geq\boldsymbol{0}, \mathbf{E}_{\boldsymbol{i}}\geq\boldsymbol{0},$$

where ${\text{||}\text{∙}\text{||}}^{2}$ stands for the element-wise quadratic norm (sum of squared elements), and $\text{≤}$ and $\geq$ for element-wise logical comparison. We call this a rank-one non-negative matrix factorization over-approximation (NMF-OA) problem as $\mathbf{K}_{\boldsymbol{i}}$ and $\mathbf{E}_{\boldsymbol{i}}$ have rank 1 and $\mathbf{K}_{\boldsymbol{i}}{\mathbf{E}_{\boldsymbol{i}}}^{\boldsymbol{T}}\geq\text{F}_{\boldsymbol{i}}\text{ }$ .

If we ignore the over-approximation constraint, our problem can be written as a rank one nonnegative matrix factorization problem:

$$\min||\mathbf{F}_{i}-\mathbf{K}_{i}\mathbf{E}_{i}^{T}||^{2} s.t. \mathbf{K}_{i}\geq0, \mathbf{E}_{i}\geq0.$$

According to Eckart-Young-Mirsky theorem (1), there is an analytical solution by singular value decomposition (SVD) of $\mathbf{F}_{i}$. Consider the following singular value decomposition of the coverage matrix $\mathbf{F}_{i}$ as

$$\mathbf{F}_{i}=\mathbf{U}\Sigma\mathbf{V}^{T}\in\mathbb{R}^{p\times L_{i}}.$$

If we let $\mathbf{U}_{1}$ denote the first left singular vector in $\mathbf{U}$, $\sigma_{1}$ be the first and largest singular value in $\Sigma$ and $\mathbf{V}_{1}$ as the first right singular vector in $\mathbf{V}$, then the truncated singular value decomposition

$${\overset{̃}{\mathbf{F}}}_{i}=\mathbf{U}_{1}\sigma_{1}\mathbf{V}_{1}^{T}$$

is the solution to the minimization problem, i.e.,

$${\overset{̃}{\mathbf{F}}}_{i}=\mathrm{argmin}_{{\overset{^}{\mathbf{F}}}_{i}}||\mathbf{F}_{i}-{\overset{^}{\mathbf{F}}}_{i}||^{2}.$$

Based on this theorem, we have our estimates of $\mathbf{K}_{\boldsymbol{i}}$ and $\mathbf{E}_{\boldsymbol{i}}$ as follows:

$\mathbf{K}_{i}=\mathbf{U}_{1}$,

$\mathbf{E}_{i}=\sigma_{1}\mathbf{V}_{1}$.

$\mathbf{K}_{i}$ and $\mathbf{E}_{i}$ are both nonnegative according to results from Perron-Frobenius theorem (2, 3) given that $\mathbf{F}_{i}$ is nonnegative matrix.

### NMF with over-approximation (NMF-OA) and Lagrangian duality

With the over-approximation constraint, we rewrite the original optimization problem by adding a constant factor as follows:

$\min\frac{1}{2}||\mathbf{F}_{i}-\mathbf{K}_{i}\mathbf{E}_{i}^{T}||^{2} such that \mathbf{F}_{i}\leq\mathbf{K}_{i}\mathbf{E}_{i}^{T}, \mathbf{K}_{i}\geq0, \mathbf{E}_{i}\geq0.$ [1]

Inspired by the work of Gillis and Glineur (4) in solving the under-approximation problem, we propose a similar optimization algorithm for NMF-OA problem using Lagrangian relaxation (5) on the over-approximation constraints.

Within imposing a Lagrangian multiplier, the Lagrangian loss function of NMF-OA can be written as

$$L(\mathbf{K}_{i},\mathbf{E}_{i},\boldsymbol{\Lambda})=\frac{1}{2}||\mathbf{F}_{i}-\mathbf{K}_{i}\mathbf{E}_{i}^{T}||^{2}+\sum_{x=1}^{L} \sum_{j=1}^{p} \boldsymbol{\Lambda}_{jx}(\mathbf{F}_{i}-\mathbf{K}_{i}\mathbf{E}_{i}^{T})_{jx} , \mathrm{where}\boldsymbol{\Lambda}=\{\boldsymbol{\Lambda}_{jx}\}\geq0.$$

Without confusion, for any matrix $\mathbf{M}$, $\mathbf{M}_{ij}$ refers to the element in $i^{th}$ row and $j^{th}$ column throughout this paper.

Note that solving the optimization problem in Eq. [1] is equivalent to solving the following problem:

$\min_{\mathbf{K}_{i},\mathbf{E}_{i}\geq0}\sup_{\Lambda\geq0}L\left( \mathbf{K}_{i},\mathbf{E}_{i},\boldsymbol{\Lambda} \right).$ [2]

This problem defined in Eq. [2] is called the primal problem. To find a solution to this primal problem is not tangible and $\sup_{\Lambda\geq0}L(\mathbf{K}_{i},\mathbf{E}_{i},\boldsymbol{\Lambda})$ is $\infty$ for $\mathbf{K}_{i}$, $\mathbf{E}_{i}$ that violates the constraints. Here we define the Lagrangian dual function as follows:

$$f(\boldsymbol{\Lambda})=\min_{\mathbf{K}_{i},\mathbf{E}_{i}\geq0}L(\mathbf{K}_{i},\mathbf{E}_{i},\boldsymbol{\Lambda}).$$

Since the loss function can be written as:

$$L\left( \mathbf{K}_{i},\mathbf{E}_{i},\boldsymbol{\Lambda} \right)=\frac{1}{2}{\mathbf{||F}_{i}+\boldsymbol{\Lambda}-\mathbf{K}_{i}\mathbf{E}_{i}^{T}||}^{2}-\frac{1}{2}{|\left| \boldsymbol{\Lambda} \right||}^{2}.$$

Given any $\boldsymbol{\Lambda}$, minimizing the Lagrangian loss function $L(\mathbf{K}_{i},\mathbf{E}_{i},\boldsymbol{\Lambda})$ is then equivalent to minimizing $||\mathbf{F}_{i}+\boldsymbol{\Lambda}-\mathbf{K}_{i}\mathbf{E}_{i}^{T}||$. $\mathbf{F}_{i}+\boldsymbol{\Lambda}$ is a non-negative matrix and this become a rank one NMF problem with solution described in the previous section. For any $\boldsymbol{\Lambda}$, the Lagrangian dual function $f(\boldsymbol{\Lambda})$ is well defined and can be solved. By application of Lagrangian duality (6), we seek an alternative to find a lower bound on the optimal NMF-OA:

$\min_{\mathbf{K}_{i},\mathbf{E}_{i}\geq0}\sup_{\Lambda\geq0}L(\mathbf{K}_{i},\mathbf{E}_{i},\boldsymbol{\Lambda})\geq\sup_{\Lambda\geq0}\min_{\mathbf{K}_{i},\mathbf{E}_{i}\geq0}L(\mathbf{K}_{i},\mathbf{E}_{i},\Lambda)=\sup_{\Lambda\geq0}f(\boldsymbol{\Lambda}).$ [3]

The right hand side of Eq. [3] ($\sup_{\Lambda\geq0}f(\boldsymbol{\Lambda})$) is called the Lagrangian dual problem as it pairs with the primal problem on the left.

Now that we are able to convert the intangible primal problem into its dual though a gap might exist because our primal problem is not convex. By solving the dual problem, we will obtain an approximate solution to the primal problem. The new dual problem requires optimization of the Lagrangian dual function $f(\boldsymbol{\Lambda})$ over a convex set $\boldsymbol{\Lambda}\geq0$. Note that -$f(\boldsymbol{\Lambda})$ is convex regardless whether the primal objective function is convex or not, but non-differentiable because the min function is not differentiable with respect to $\boldsymbol{\Lambda}$. We will consider some sub-gradient optimization method to iteratively estimate $\boldsymbol{\Lambda}$.

### Updating multiplier $\boldsymbol{\Lambda}$ - Subgradient optimization

The rest of the work is to update $\boldsymbol{\Lambda}$ using subgradient optimization. Subgradient optimization is often used for non-differentiable convex objective functions (7, 8). A subgradient is a generalized term of gradient as defined below:

**Definition: Subgradient**

*If* $g:U\mathbb{\to R}$ *is a convex function defined on a convex open set in* $\mathbb{R}^{n}$*, a vector* $v$ *in that space is called a subgradient at a point* $x_{0}$ *in* $U$ *if for any* $x$ *in* $U$ *one has*

$$g(x)-g(x_{0})\geq v^{T}\cdot(x-x_{0}).$$

Note that in our dual problem, we can see that $\mathbf{K}_{i}\mathbf{E}_{i}^{T}-\mathbf{F}_{i}$ is actually a subgradient of -$f(\boldsymbol{\Lambda})$. Based on the subgradient optimization idea in (8), we can update $\boldsymbol{\Lambda}$ by negative subgradient as follows:

$$\boldsymbol{\Lambda}\leftarrow\max(0,\boldsymbol{\Lambda}-\alpha_{n}(\mathbf{K}_{i}\mathbf{E}_{i}^{T}-\mathbf{F}_{i})),$$

where $\alpha_{n}$ is the selected sequence of step sizes for the $n^{th}$ iteration. There are many choices of step sizes $\alpha_{n}$ selection where the convergence has been proved ^(^9, 10) provided $\alpha_{n}$ satisfies:

$$\alpha_{n}\geq0,\lim_{n\to\infty}\alpha_{n}=0,\sum_{n=1}^{\infty} \alpha_{n}=\infty.$$

In our algorithm we adopt a nonsummable diminishing step size that $\alpha_{n}=\frac{1}{\sqrt{n}}$. The iterative procedure, to be referred to as below, can be summarized as follows:

1. Initializing $\boldsymbol{\Lambda}=0$ and $n=1$;
2. Updating $\mathbf{K}_{i}$ and $\mathbf{E}_{i}$ by NMF of $\mathbf{F}_{i}+\boldsymbol{\Lambda}$;
3. Updating $\boldsymbol{\Lambda}$ by $\max(0,\boldsymbol{\Lambda}-\frac{1}{\sqrt{n}}(\mathbf{K}_{i}\mathbf{E}_{i}^{T}-\mathbf{F}_{i}))$;
4. Stop if n is greater than a pre-specified maximum of iteration rounds (by default 100); Otherwise, $n=n+1$ and go to step 2.

The subgradient method is usually practiced without rigorous stopping criterion (11) because the value in determination of the stopping criteria often goes to zero very slowly. In our application to the read counts matrix, usually around 100 iterations will yield satisfactory results and running more iterations does not help much in the output of the algorithm.

## Gene and transcript region filtering for DegNorm

We apply a loose filtering criterion for which regions of a transcript and which genes to be included in the DegNorm algorithm. A region with extremely low coverage scores from all samples is less informative for detection of degradation or baseline selection. Define

$$C_{i}=\left\{ x\in\left\{ 1,2,\cdots,L_{i} \right\}:\max_{j} f_{ij}\left( x \right)>0.1 \max_{j,x} f_{ij}\left( x \right) \right\}, \mathrm{where} j=1,\cdots,p.$$

DegNorm is only performed in the subset transcript regions defined by $C_{i}$ provided that length of $C_{i}$is at least 50, and the total coverage of each sample on $C_{i}$is >0. The main motivation of this criterion is to prevent regions that have extremely low coverage score relative to other regions but with similar flat shape from being selected as the baseline. The genes excluded from DegNorm typically have a spike (<50 bp) in coverage curve in some sample(s) while having flat curves in all other regions. For these genes, we adjust the read count based on the average degradation index score of the corresponding sample.

## RNA-seq data processing and read coverage score calculation

All datasets analyzed in this paper were obtained from the NCBI Sequence Read Archive (SRA) or Gene Expression Omnibus (GEO). We mapped the raw data to respective reference genomes (hg19 for Homo Sapiens, Zv9 for Danio rerio and the ERCC spike-in sequences http://tools.invitrogen.com/downloads/ERCC92.fa) using TopHat (v2.0.14) (12) under the default setting. For paired end data, only concordant and uniquely mapped pairs were kept for read count and coverage score calculation. For the single end data, only uniquely mapped reads were used in the downstream analysis.

As many genes may have multiple splicing forms, we define the total transcript as the concatenation of all exons. We used UCSC genome annotation file (.gtf) to extract the exons locations of each gene. For any gene, the read coverage score at a given base pair is defined as the total number of fragments aligned to it. For the single end data, this coverage score is simply defined as the number of read aligned to each genomic location. For each paired-end read, alternative splicing makes it complicated to calculate the coverage score. There are four different situations: 1) If the two ends overlap with each other, then we add 1 to the coverage score in the exon region(s) that corresponds to the assembly of two ends. 2) If the two ends do not overlap but both are mapped to the same exon, then we add 1 to the coverage score in the exon region between the two ends. 3) If the two ends do not overlap, but aligned to two neighboring exons in the total transcript, then we add 1 to the coverage score in the two exon regions between the two ends. 4) If the two ends do not overlap, but aligned to two non-neighboring exons, we first add 1 to the coverage score in the two aligned exons from the end start positions to the boundaries of the exons. We assume the fragment maximum length is 300 bp. If the total length of the two paired ends plus the exon(s) between the two end <= 300 bp, we add 1 to the coverage score of all exon regions between the two ends. Otherwise this paired end read does not contribute to the coverage score in the exons between the two aligned exons.

## PCR normalization and log fold-change calculation

In the analysis of SEQC data, we utilized the real-time PCR (polymerase chain reaction) results as the gold standard for comparison. The RT-qPCR assay is method to quantify mRNA expression using fluorescent dyes. By creation of cDNA using reverse transcription of mRNA, RT-qPCR provides $C_{T}$ (threshold cycle) values defined as the number of PCR cycle at which the fluorescent signal reaches the preset threshold.

We considered two sets of PCR results for SEQC data, one from Tagman data stored in R package ‘seqc’, and the other from SEQC Consortium of 20,801 PCR verified genes. For set-1, we were able to find the calculated log fold change from the RUVg paper. While for set-2, we only have the raw $C_{T}$ values and normalization is necessary to use the PCR results as gold standard for further comparison.

Using housekeeping or negative control genes is one way of normalizing PCR data and related methods are widely applied in the literatures (13). Here we use geNorm (14) method implemented in the R package NormqPCR (15) to select negative control genes for set-2 PCR data.

Let $G$ denote the selected set of negative control genes from geNorm method, the fold change for gene $i$ is defined as in (16):

$fold change=2^{-\Delta\Delta C_{T}}$.

Then the $\log_{2}$ fold change between condition A and B is defined as:

$\log_{2} fold change=-\Delta\Delta C_{T}$,

$$-\Delta\Delta C_{T}=(C_{T}(A)-(\prod^{g\in G} C_{T}^{g}(A))^{\frac{1}{|G|}})-(C_{T}(B)-(\prod^{g\in G} C_{T}^{g}(B))^{\frac{1}{|G|}})$$

Simply put, the raw $C_{T}$ values are subtracted by the geometric mean of the $C_{T}$ values from selected housekeeping genes in each sample and the differences in the normalized $C_{T}$ values are considered as $\log_{2}$ fold change.

1. **Simulation details**

The simulated data is in two-condition comparison setting: 4 control vs. 4 treatment samples in three different degradation settings. Each sample was simulated with a random sequencing depth of 40-60 million reads. Each setting contains 20,000 genes, among which 80% were subject to degradation, 5% were chosen to be up-regulated and 5% for down-regulated. In the first setting, for a gene selected for degradation, 3, or 4 or 5 samples out of the 8 were randomly chosen for degradation whereas in the second, either all four samples from the control or treatment were randomly chosen for degradation. In the third setting, for each gene to degrade, two control samples were randomly selected for degradation with the same marginal distribution while all treatment samples underwent degradation but with different expected severity. The simulation details are described below.

The simulation consists of two parts. The first part is to simulate the latent read counts matrix $\boldsymbol{Y}$ from the negative binomial distributions. The second part is to simulate the reads distribution given the latent count for each gene within each sample. If one gene is selected for degradation, we will simulate the reads distribution according to the simulated degradation pattern.

**4.1 Simulation of latent reads count**

We simulate the latent reads count from negative binomial model. To mimic the real data, we randomly sample the fitted mean ($\mu$) and dispersion parameters ($\phi$) estimated from SEQC-AB dataset. The simulation of latent read counts contains the following steps.

1. For a given gene $g$ in the control samples, the abundance level $\lambda_{gc}$ for gene $g$ was randomly drawn from the fitted mean values for SEQC-AB data using edgeR, for $g=1,...,N$, where $N=20000$.
2. For the treatment samples, the abundance level was set as

$$d_{g}=\left\{ \begin{aligned} 1.5+\gamma_{g}, &if gene g is up regulated \\ \frac{1}{1.5+\gamma_{g}}, &if gene g is down regulated \\ 1, &if gene g is nondifferentially expressed \end{aligned} \right.,$$

and $\gamma_{g}\sim exp(1).$

1. Let $S_{j}$ be a sequencing depth factor for sample $j$ and $S_{j}={10}^{7}U_{j}$where $U_{j} \sim U(4, 6)$. Thus the mean parameter for gene $g$ in sample $j$ is given by

$$\mu_{gj}=\frac{\lambda_{gj}}{\sum_{i=1}^{N} \lambda_{ij}}S_{j},$$

where $\lambda_{gj}=\lambda_{gc}$ if $j^{th}$ sample is assigned to the control group, or $\lambda_{gj}=\lambda_{gt}$ otherwise.

1. The latent read counts is generated from a negative binomial distribution

$$Y_{gj}\sim NB(\mu_{gj}, \phi_{g}),$$

where the over-dispersion parameter $\phi_{g}$ is randomly drawn from the estimated dispersion parameter value in the SEQC-AB data.

Following procedure (1)-(4) listed above, we are able to generate read counts matrix $\boldsymbol{Y}$ containing both differentially expressed and non-differentially expressed genes from negative binomial models. Based on $\boldsymbol{Y}$, we can simulate reads coverage curves as described in the next section.

- 1. **Simulation of reads distribution**

For simplicity we do not consider alternative splicing in this simulation. We first simulate the reads start positions, based on which we calculate the read coverage curve by counting the number of aligned fragments that cover each given position on the transcript (assuming the fragment length of each read is 200 bp).

We draw effective transcript length ($L_{g}$) for gene $g$ from the empirical distribution of human reference genome (hg19), where $L_{g}$ is the summed length of all exons. The reads start positions are assumed to follow a normal mixture model with $K$ components:

$$f\left( x \right)= \sum_{k=1}^{K} \frac{1}{K}f_{k}(x;\mu_{k}, \sigma) ,$$

where $f_{k}(x;\mu_{k},\sigma)$ is the Gaussian density function for the $k^{\mathrm{th}}$ component with mean $\mu_{k}$ and standard deviation $\sigma$ ($\sigma$ is set to be 200 bp in the simulation). Note that this normal mixture is defined in integer positions of the full length of given transcript, i.e., from $1$ to $L_{g}$. We set the weights of normal mixture as equal for each component, the number of components $K$ be the greatest integer less than $\frac{L_{g}}{\sigma}$, and the mean $\{\mu_{k}\}$ be uniform from $U(0,L_{g}-\sigma)$. Although ideally the expected reads coverage curve is flat in the middle and the distribution of reads start position is expected to be uniform, the actual curve varies substantially and typically has multiple modes in the simulated data. Thus using a normal mixture in the simulation would present a good mimic of observed variation in the real data.

- 1. **Simulation of transcript degradation**

Degradation may happen at any regions of the transcript in the real data. Here we consider the most commonly observed $5^{'}$ degradation for simplicity. To simulate the degradation pattern, we consider applying an acceptance rate $p(x)$ to each simulated read given the read starting position $x$ where $0\leq p(x)\leq1$. By varying the shape of $p(x)$, we can control the direction and severity of degradation. Generally any non-negative function could be used to serve for this purpose. In our simulation, we decided to use the following function $p(x, \sigma_{g})$ to calculate the acceptance probability to simulate the degradation pattern for its monotone increasing pattern resembles the attenuated degradation from $5^{'}$ to $3^{'}$ end:

$$p(x, \sigma_{g})= \frac{1}{F\left( 1.5;0,\sigma_{g} \right)*F\left( 2;0,\sigma_{g} \right)}F(\frac{2x}{L_{g}};0,\sigma_{g}).$$

We let $L_{g}$ denote the effective transcript length, $x$ be the read starting position and $F(x;0,\sigma_{g})$ be the cumulative distribution function of a log normal distribution with mean 0 and standard deviation $\sigma_{g}$ both on the log scale.

For each read, the start position $x$ is first simulated from the normal mixture. Each read has a probability $p(x)$ to be accepted (or $1-p(x)$ to be discarded or degraded). We can tune $\sigma_{g}$ to attain the desired the degradation pattern for each gene within each sample. With $\sigma=0.5$, we have the severe degradation while with larger $\sigma=1.5$, the degradation is controlled to be within a minimum level. In our simulations when we range $\sigma$ between 0.5 and 1.5 the resulting pattern of degradation is (from right to left):

SM_Figure 1 Acceptance rates of simulated latent reads

Given the latent counts ($Y_{gj}$) and degradation pattern ($p_{gj}(x))$, the simulation of degraded read counts ($X_{gj}$) and reads coverage curves can be summarized as follows:

1. Initialize $X_{gj} = 0$ and $i=1$;
2. Simulate read starting position $x_{i}\in[1,2,\ldots,L_{g}-199] , x_{i}\sim f(x)$;
3. Generate a random number $r$, $r\sim U(0,1)$ . If $r\leq p_{gj}(x_{i})$, this read is kept and counted $X_{gj} = X_{gj} + 1$; otherwise this read is considered as degraded.
4. If $i < Y_{gj}$, go to step (1); otherwise stop.

Altering the shape of $p_{gj}(x)$, we can adjust the degradation pattern. Then for gene $g$ in sample $j$, we have the simulated observed count $X_{gj}$, latent count $Y_{gj}$ along with reads starting positions $x_{i}$, $i=1,\cdots,Y_{gj}$. The reads coverage curve can be calculated accordingly.

- 1. **Simulation settings**

Now we provide more details about the degradation setting in simulation II-IV. For genes in samples unaffected by degradation, $p(x)=1$ for all $x\in(0,L_{g})$ and consequently $X_{gj} = Y_{gj}$. If a gene is chosen to degrade, we set specific patterns in $p(x,\sigma)$ to approximate the natural process of mRNA degradation by tuning $\sigma$. The abundance level, the gene-wise dispersion and the latent counts simulation for each gene are independent from each other.

**Simulation setting-II**: Number of genes simulated $N=20000$. 10% of *N* genes are set to be differentially expressed with 5% up-regulated and 5% down-regulated genes. 80% of the genes are randomly chosen to be degraded. Both differentially and non-differentially expressed genes are subject to the same probability for degradation. For a gene selected for degradation, a random number $k$ samples ($k\sim\mathrm{Uniform}[3, 4, 5]$) out of 8 samples are randomly selected for degradation at random level with acceptance rate $p(x, \sigma_{i}),$ where $\sigma_{i}\in U(0.5, 1.5)$ $i=1,\cdots,k$. For this simulation, degradation effect is random among both treatment and control samples and differs between genes.

**Simulation setting-III**: Number of genes simulated $N=20000$. 10% of *N* genes are set to be differentially expressed with 5% up-regulated and 5% down-regulated genes. 80% of the genes are randomly chosen to be degraded. For each degraded gene, one condition is randomly selected (treatment or control) to be degraded. For example, if the treatment samples are selected to be degraded, the degradation levels for the treatment samples are randomly drawn with acceptance rate $p(x, \sigma_{ti})$ where $\sigma_{ti}\sim U(0.5, 1.5),$ $i=1,\cdots,4$. In this simulation, degradation effect has systematic difference between the two conditions for any gene subject to degradation while the overall degradation levels are similar between the two conditions.

**Simulation setting-IV**: Number of genes simulated $N=20000$. 10% of N genes are set to be differentially expressed with 5% up-regulated and 5% down-regulated genes. 80% of the genes are randomly chosen to be degraded. For each degraded gene, all treatment samples are set to be degraded and the degradation level increases from Trt1 to Trt4 where $\sigma_{ti}\sim U(0.5, 1.5)$and $\sigma_{t1}>\sigma_{t2}>\sigma_{t3}>\sigma_{t4}$ (i.e., we choose the order statistics). For control samples, 2 samples are randomly selected to degrade with $\sigma_{ci}\sim U\left( 0.5, 1.5 \right), i = 1, 2$.

**Supplementary References**

1. Eckart C, Young G. The approximation of one matrix by another of lower rank. Psychometrika. 1936;1(3):211-8.

2. Frobenius FG. Über Matrizen aus nicht negativen Elementen. 1912.

3. Perron O. Zur theorie der matrices. Mathematische Annalen. 1907;64(2):248-63.

4. Gillis N, Glineur F. Using underapproximations for sparse nonnegative matrix factorization. Pattern recognition. 2010;43(4):1676-87.

5. Boyd S, Vandenberghe L. Convex optimization: Cambridge university press; 2004.

6. Fisher ML. An applications oriented guide to Lagrangian relaxation. Interfaces. 1985;15(2):10-21.

7. Held M, Wolfe P, Crowder HP. Validation of subgradient optimization. Mathematical programming. 1974;6(1):62-88.

8. Shor NZ. Minimization methods for non-differentiable functions: Springer Science & Business Media; 2012.

9. Anstreicher KM, Wolsey LA. Two “well-known” properties of subgradient optimization. Mathematical Programming. 2009;120(1):213-20.

10. Bazaraa MS, Sherali HD. On the choice of step size in subgradient optimization. European Journal of Operational Research. 1981;7(4):380-8.

11. Boyd S, Xiao L, Mutapcic A. Subgradient methods. lecture notes of EE392o, Stanford University, Autumn Quarter. 2003;2004:2004-5.

12. Trapnell C, Williams BA, Pertea G, Mortazavi A, Kwan G, van Baren MJ, et al. Transcript assembly and quantification by RNA-Seq reveals unannotated transcripts and isoform switching during cell differentiation. Nat Biotechnol. 2010;28(5):511-5.

13. Livak KJ, Schmittgen TD. Analysis of relative gene expression data using real-time quantitative PCR and the 2(-Delta Delta C(T)) Method. Methods. 2001;25(4):402-8.

14. Vandesompele J, De Preter K, Pattyn F, Poppe B, Van Roy N, De Paepe A, et al. Accurate normalization of real-time quantitative RT-PCR data by geometric averaging of multiple internal control genes. Genome Biol. 2002;3(7):RESEARCH0034.

15. Perkins JR, Dawes JM, McMahon SB, Bennett DL, Orengo C, Kohl M. ReadqPCR and NormqPCR: R packages for the reading, quality checking and normalisation of RT-qPCR quantification cycle (Cq) data. BMC Genomics. 2012;13:296.

16. Schmittgen TD, Livak KJ. Analyzing real-time PCR data by the comparative C(T) method. Nat Protoc. 2008;3(6):1101-8.
