# Supplementary material for: DegNorm: normalization of generalized transcript degradation improves accuracy in RNA-seq analysis
Source: Genome Biol. 2019 Apr 16;20:75. doi: 10.1186/s13059-019-1682-7 (PMC6466807; doi:10.1186/s13059-019-1682-7)
Supplement: Supplementary file 2 — Supplementary figures and figure legends. (PDF 69270 kb) [file 13059_2019_1682_MOESM2_ESM.pdf]

Supplementary Figure Legends

Figure S1 Heatmaps of pairwise Pearson correlation and scatter plot of DI scores between samples (a-f) Shown are results for DI scores for SEQC-AA, SEQC-AB, PBMC-S01, GBM, DLPFC-Br1729 and Breast Tumor data respectively. SEQC-AB shows a strong between-condition correlation in degradation pattern, so does GBM R10 vs. R6+R4. (**g**-**i**) Scatter plot of DI scores between samples of same RIN or different RIN in GBM data.

Figure S2 Choice of empirical controls genes and factors in RUVg normalization method may dramatically affect differential expression analysis (a-c) Coefficient of variation (CV) vs. mean read count (in log scale) from RUVg method for GBM R10 vs. R4, PBMC-S01 and SEQC-AB respectively. The mean counts for each data were scaled to 0-1 range by a linear transformation (i.e., $(X_i - min_j(X_j))/max_j\,(X_j)$. Compared are results when different number of empirical genes were used in estimation of the unwanted variation. For each data set, we excluded L most significant genes and used the remaining as empirical controls to estimate the unwanted variation factor for L=1000, 3000, 5000, 8000 and 10000 respectively. In all four data sets presented, the first factor from the factor analysis was used in the estimation of unwanted variation. The CV curve was smoothed using the built-in smoothing spline function in R. In the GBM R10 vs. R4 and PBMC-S01 comparison, the CV curves were extremely sensitive to the choice of L, while not for the SEQC-AB data. **(d-f)** Empirical CDF of the p-value from DE analysis for each data set under each setting as specified in (**a**-**c**). Again ECDF curves show sensitivity to the choice of empirical control genes in GBM R10 vs. R4 , PBMC-S01 and SEQC-AB data. (**g, h**) ECDF of the p-value for GBM-R10 vs. R4 and PBMC-S01 data when the second factor from factor analysis

was used to estimate the unwanted variation for $L = 5000$. The curves were dramatically changed compared to results in (**e**, **f**) where the first factor was used.

Figure S3 Comparing different global normalization methods in Differential expression analysis (**a**-**e**) Comparison of ECDF plots of the p-values of DE analysis under different normalization methods including DegNorm, upper quartile (UQ), trimmed mean of m-values(TMM), relative log expression (RLE) and total read count (TC). The curves from the latter four global normalization methods are highly consistent and almost overlap perfectly for all the six comparisons under investigation.

Figure S4 DE analysis of DLPFC data (**a**) ECDF of p-values for DE analysis of DLPFC subject Br1729 mRNA-seq data T0+T15 vs T30+T60. (**b**) ECDF of p-values for DE analysis of DLPFC subject Br1729 mRNA-seq (T0+T15+T30+T60) vs. Ribo-Zero-seq data (T0+T15+T30+T60).

Figure S5 Results for Simulations II-IV (**a-f**) Box plot of true vs. estimated DI scores for each simulation. Condition A and B stand for control and treatment samples respectively. (**g-l**) Heatmap of Pearson correlation of true vs. estimated DI scores between samples.

Figure S6 Results for Simulations II-IV (**a**-**d**) Scatter plot of normalized read count vs. latent read count in log2 scale with diagonal line imposed for Simulation III under different normalization methods. The latent count was adjusted for sequencing depth using UQ method. R^2 is the regression coefficient of determination. (**e**-**h**) Same as (**a**-**e**) but for Simulation IV.

Fig S1

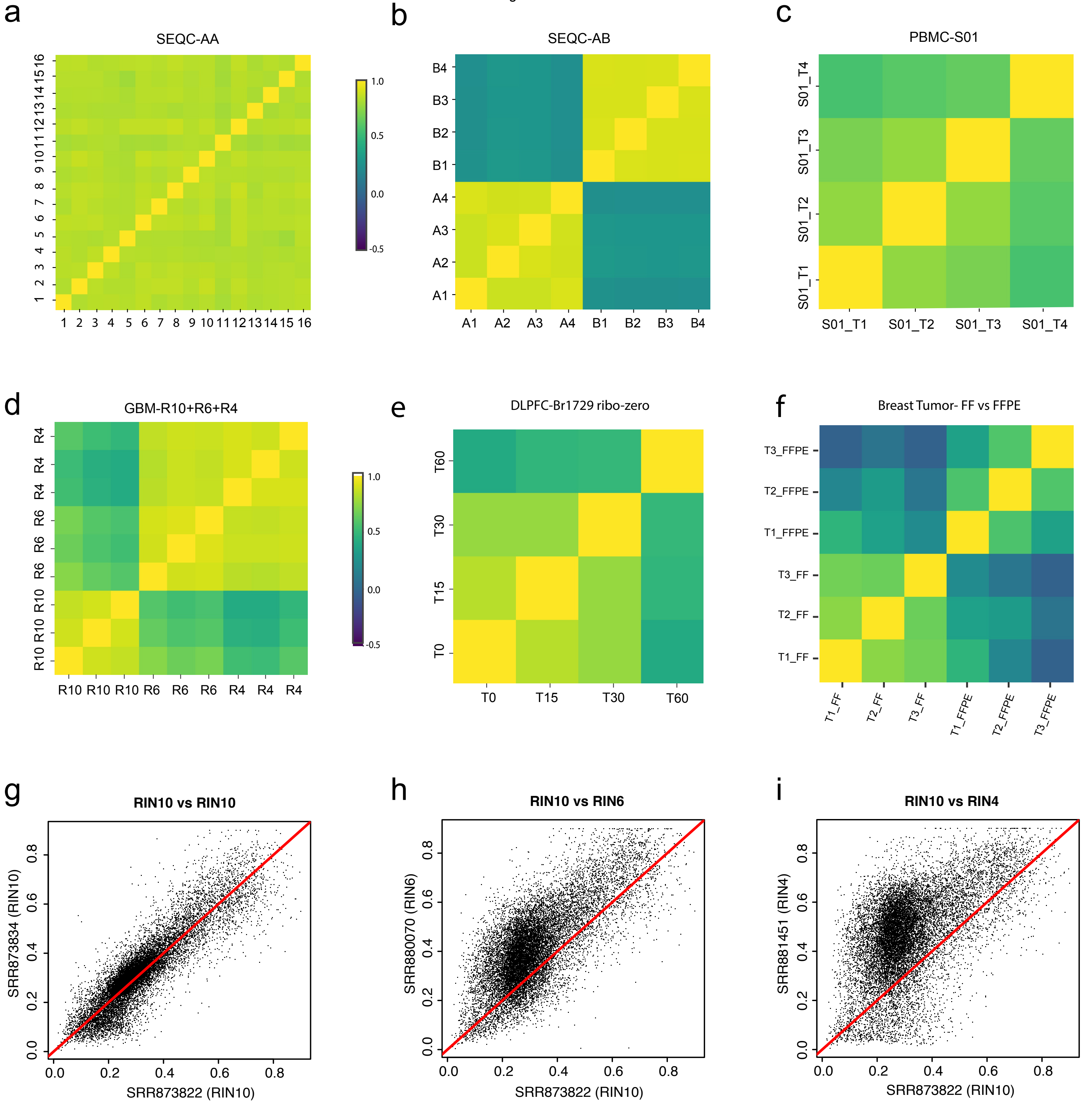
a
SEQC-AA
1 2 3 4 5 6 7 8 9 10 11 12 13 14 15 16
1.0
0.5
0.0
-0.5
b
SEQC-AB
A1 A2 A3 A4 B1 B2 B3 B4
c
PBMC-S01
S01_T1 S01_T2 S01_T3 S01_T4
d
GBM-R10+R6+R4
R10 R10 R10 R6 R6 R6 R4 R4 R4
e
DLPFC-Br1729 ribo-zero
T0 T15 T30 T60
f
Breast Tumor- FF vs FFPE
T1_FF T2_FF T3_FF T1_FFPE T2_FFPE T3_FFPE
g
RIN10 vs RIN10
SRR873834 (RIN10)
SRR873822 (RIN10)
h
RIN10 vs RIN6
SRR880070 (RIN6)
SRR873822 (RIN10)
i
RIN10 vs RIN4
SRR881451 (RIN4)
SRR873822 (RIN10)
0.0 0.2 0.4 0.6 0.8

Fig S2

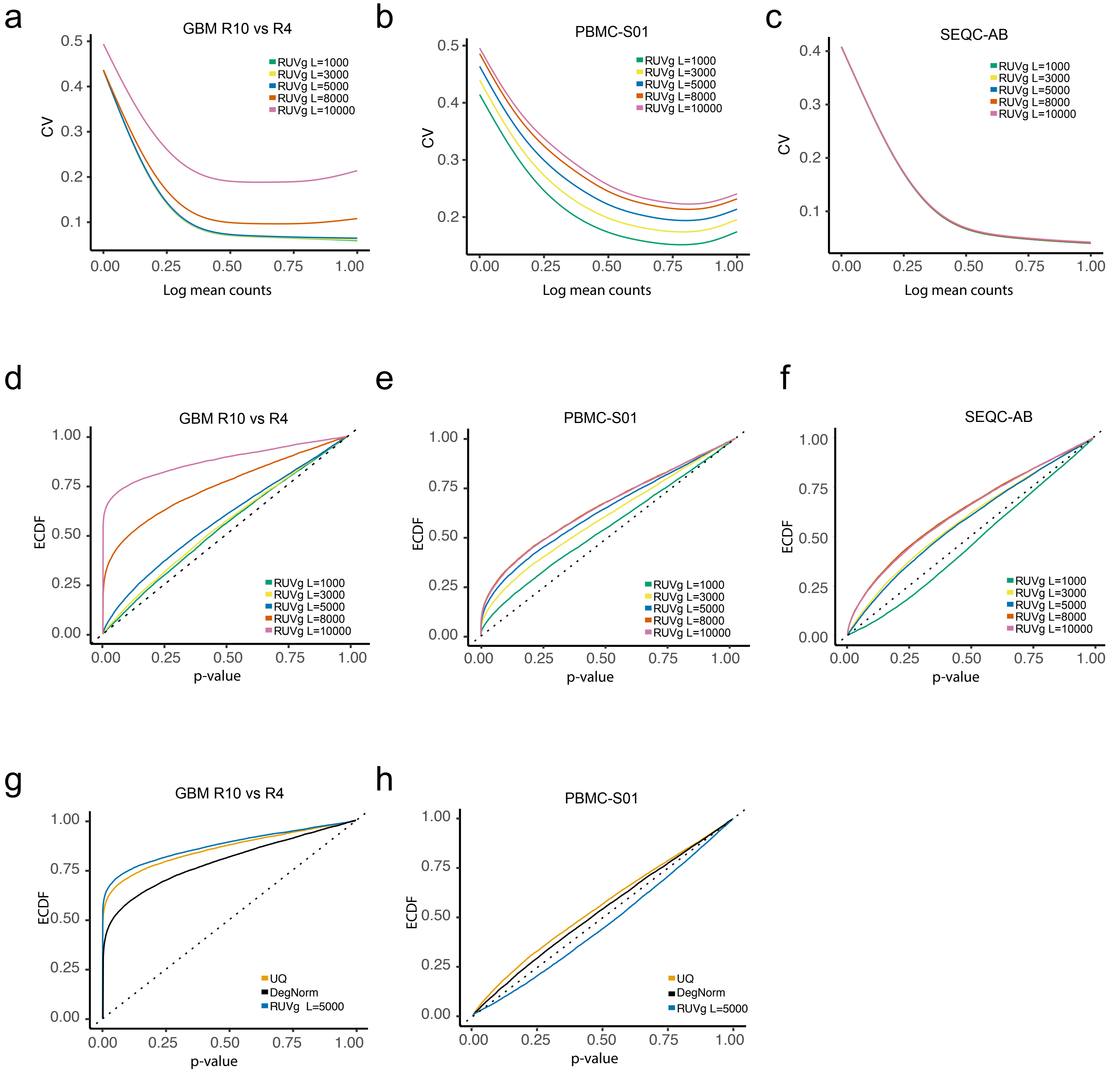

a
GBM R10 vs R4
CV
Log mean counts
RUVg L=1000
RUVg L=3000
RUVg L=5000
RUVg L=8000
RUVg L=10000
b
PBMC-S01
CV
Log mean counts
RUVg L=1000
RUVg L=3000
RUVg L=5000
RUVg L=8000
RUVg L=10000
c
SEQC-AB
CV
Log mean counts
RUVg L=1000
RUVg L=3000
RUVg L=5000
RUVg L=8000
RUVg L=10000
d
GBM R10 vs R4
ECDF
p-value
RUVg L=1000
RUVg L=3000
RUVg L=5000
RUVg L=8000
RUVg L=10000
e
PBMC-S01
ECDF
p-value
RUVg L=1000
RUVg L=3000
RUVg L=5000
RUVg L=8000
RUVg L=10000
f
SEQC-AB
ECDF
p-value
RUVg L=1000
RUVg L=3000
RUVg L=5000
RUVg L=8000
RUVg L=10000
g
GBM R10 vs R4
ECDF
p-value
UQ
DegNorm
RUVg L=5000
h
PBMC-S01
ECDF
p-value
UQ
DegNorm
RUVg L=5000

Fig S3

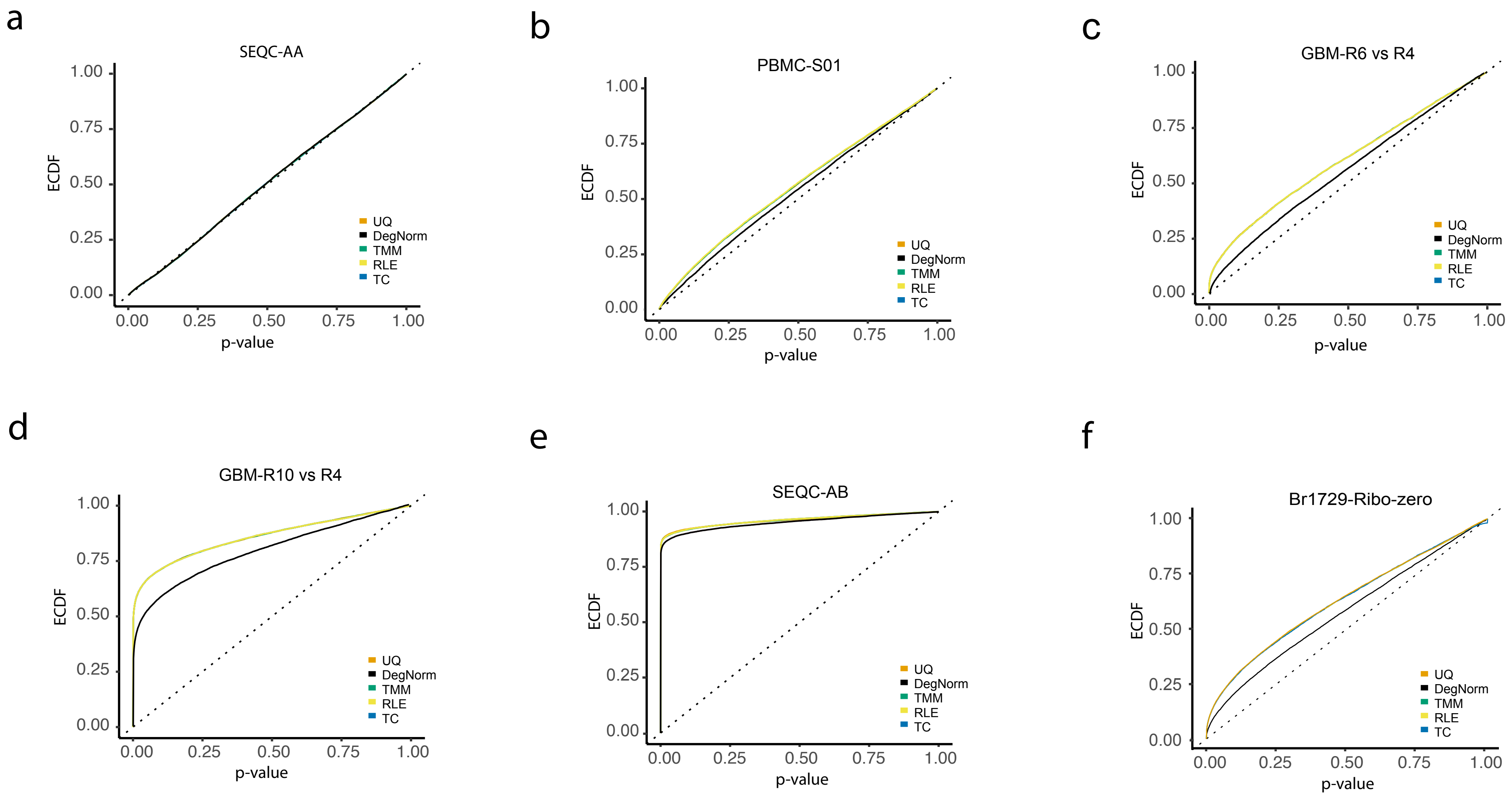

a
SEQC-AA
b
PBMC-S01
c
GBM-R6 vs R4
d
GBM-R10 vs R4
e
SEQC-AB
f
Br1729-Ribo-zero
ECDF
p-value
0.00
0.25
0.50
0.75
1.00
UQ
DegNorm
TMM
RLE
TC

Fig S4

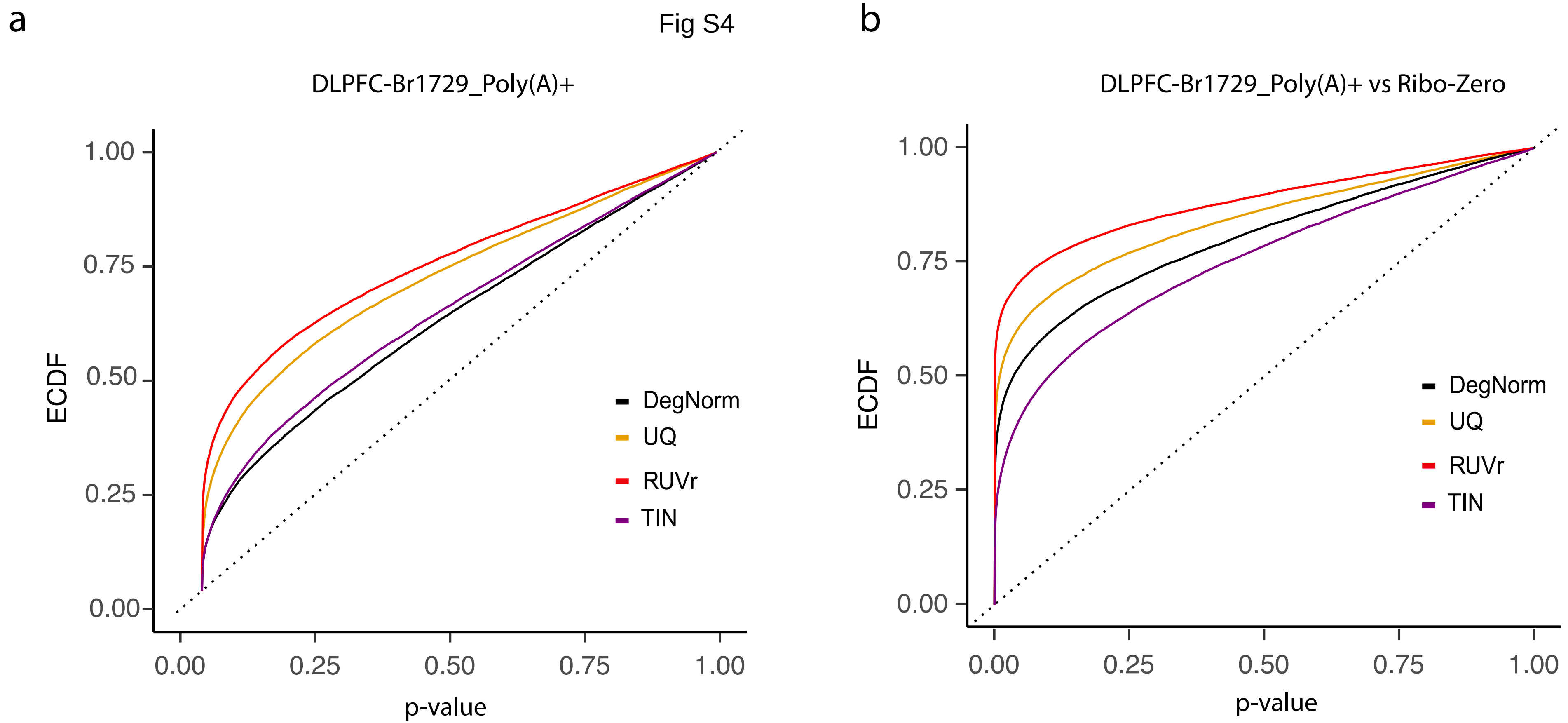
a
DLPFC-Br1729_Poly(A)+
ECDF
1.00
0.75
0.50
0.25
0.00
p-value
0.00
0.25
0.50
0.75
1.00
DegNorm
UQ
RUVr
TIN
b
DLPFC-Br1729_Poly(A)+ vs Ribo-Zero
ECDF
1.00
0.75
0.50
0.25
0.00
p-value
0.00
0.25
0.50
0.75
1.00
DegNorm
UQ
RUVr
TIN

Fig S5

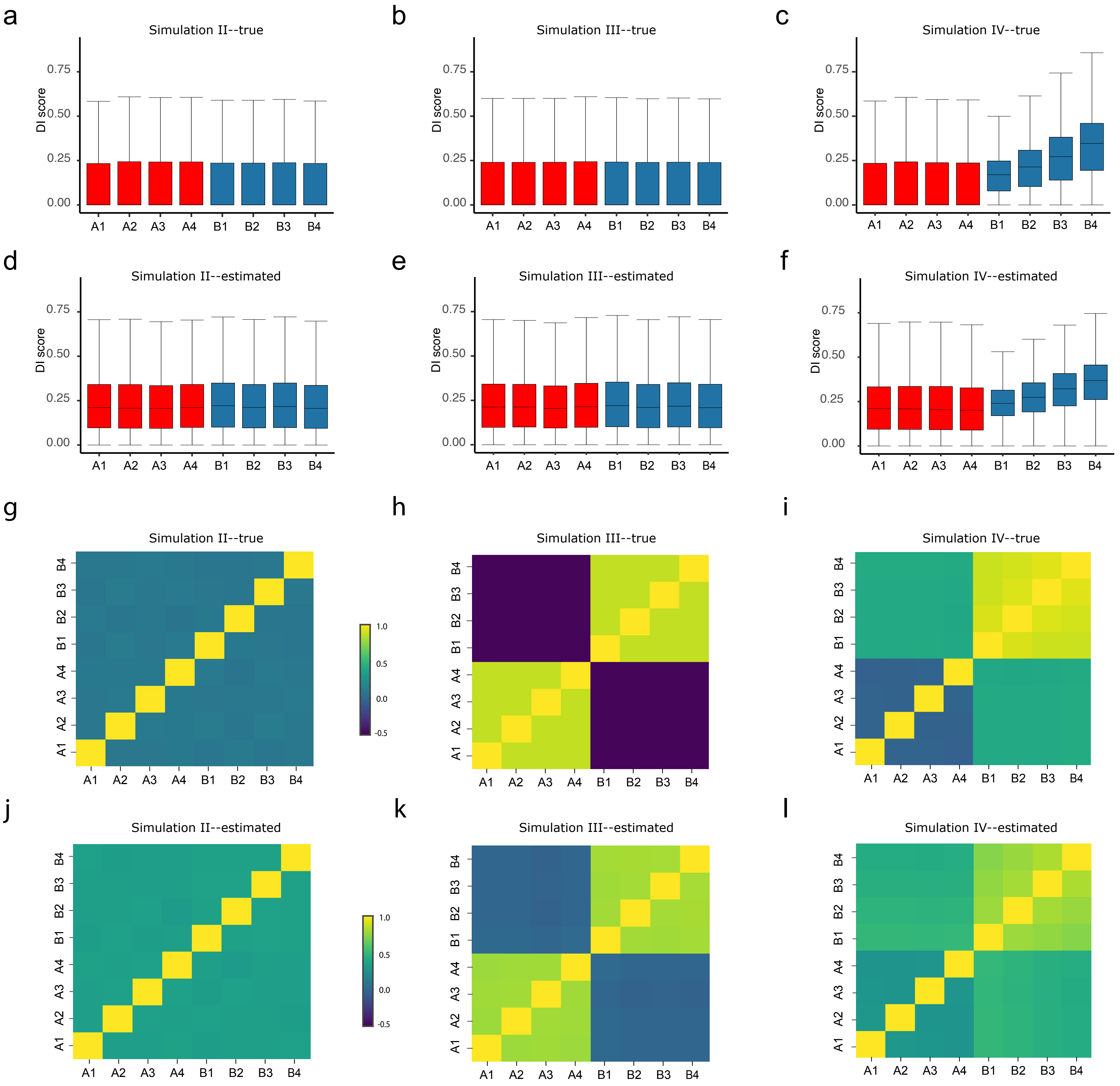
a
Simulation II--true
b
Simulation III--true
c
Simulation IV--true
DI score
0.00
0.25
0.50
0.75
A1
A2
A3
A4
B1
B2
B3
B4
d
Simulation II--estimated
e
Simulation III--estimated
f
Simulation IV--estimated
g
Simulation II--true
h
Simulation III--true
i
Simulation IV--true
1.0
0.5
0.0
-0.5
j
Simulation II--estimated
k
Simulation III--estimated
l
Simulation IV--estimated

Fig S6

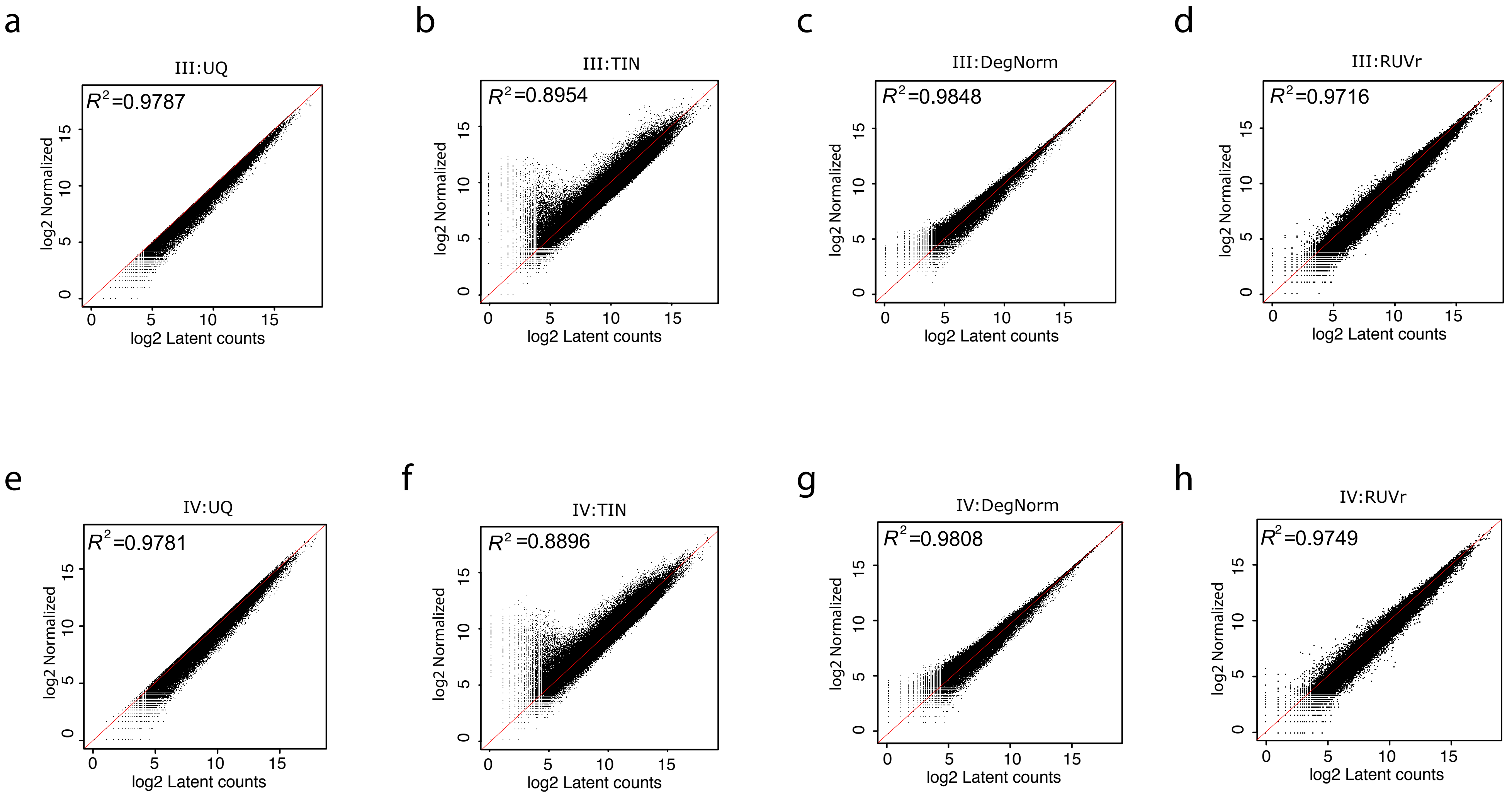
a
III:UQ
R^2=0.9787
log2 Normalized
log2 Latent counts
b
III:TIN
R^2=0.8954
log2 Normalized
log2 Latent counts
c
III:DegNorm
R^2=0.9848
log2 Normalized
log2 Latent counts
d
III:RUVr
R^2=0.9716
log2 Normalized
log2 Latent counts
e
IV:UQ
R^2=0.9781
log2 Normalized
log2 Latent counts
f
IV:TIN
R^2=0.8896
log2 Normalized
log2 Latent counts
g
IV:DegNorm
R^2=0.9808
log2 Normalized
log2 Latent counts
h
IV:RUVr
R^2=0.9749
log2 Normalized
log2 Latent counts
