# Supplementary material for: DegNorm: normalization of generalized transcript degradation improves accuracy in RNA-seq analysis
Source: Genome Biol. 2019 Apr 16;20:75. doi: 10.1186/s13059-019-1682-7 (PMC6466807; doi:10.1186/s13059-019-1682-7)
Supplement: Supplementary file 6 — Review history. (DOCX 53 kb) [file 13059_2019_1682_MOESM6_ESM.docx]

Review History

**First round of review**

**Reviewer 1**

**Are the methods appropriate to the aims of the study, are they well described, and are necessary controls included?**
Yes

**Are the conclusions adequately supported by the data shown?**

Yes

**Are sufficient details provided to allow replication and comparison with related analyses that may have been performed?**

Yes

**Does the method perform better than existing methods (as demonstrated by direct comparison with available methods)?**

Yes

**Is the method likely to be of broad utility? Is any software component easy to install and use?**

Yes. The code is available on GitHub, and the authors are "working on a software tool to automate the entire pipeline" (cited from their paper).

**Is the paper of broad interest to others in the field, or of outstanding interest to a broad audience of biologists?**

**Yes**: Proper normalization is a critical pre-processing step in any RNA-seq data analysis. The proposed method has shown clear advantage over existing methods.

**Comments to author:**

The authors proposed a novel normalization method for RNA-seq data that takes care of the degradation effect. Interestingly, their definition of degradation effect not only refers to the mRNA degradation, but also the alternative splicing, etc. This clever way bypasses the difficulty of deconvoluting the mRNA degradation effect and alternative splicing, which is virtually impossible, and gives a uniformed and easy-to-use method for normalizing individual genes. Figure 6 clearly demonstrates the very impressive performance of their method. The correction of degradation from the left panel to the right panel, especially considering the nonparametric shape of the mRNA degradation and the complicated alternative splicing pattern, seems almost impossible, but the proposed method works almost perfectly.

The algorithm they propose is solid and effective. The authors carefully pick up four real datasets, each with its own characteristics, to demonstrate different aspects of the performance of their method. Their method consistently outperforms existing methods on all four datasets. They also carefully studied their method and compared with others on simulation data.

The pipeline, if made publicly available and user friendly, will be very widely used. I expect high impact of their method.

The paper is very well written. All the real datasets have been thoroughly analyzed and the discussion about the comparison is fair and sound. I have no major concerns, and all my comments/suggestions below should be considered minor.

1. In the description of methods, the authors consider all read counts along "expressed eons in the sequential order". How about junction reads (That is, reads that connecting exons)? Did the authors just connect all exons into a large transcript (then how about alternative splicing?) or did the authors just ignore all junction reads? Including junction reads uses more information and hence may increase power, but it will also complicate things. Whatever way the author was using should be fine and does not need to be changed, but it is a good idea to describe it a bit more clearly.

2. Since Finotello et al (21) proposes a simple method for reducing degradation effect, is it possible to compare the proposed method with theirs?

3. The authors do not clearly describe how they did DE analysis after normalizing the data using DegNorm. It seems that they used edgeR (I learned this from the legend of Figure 8)?

4. There is a typo on line 34 of the page before "Figure Legends": "prinounced" should be "pronounced".

**Reviewer 2**

**Are the methods appropriate to the aims of the study, are they well described, and are necessary controls included?**
Yes, but more data and analyses are required.

**Are the conclusions adequately supported by the data shown?**

Not entirely, see my comments to the authors.

**Are sufficient details provided to allow replication and comparison with related analyses that may have been performed?**

The DegNorm code should be made publicly available.

**Does the method perform better than existing methods (as demonstrated by direct comparison with available methods)?**

**No**: Claim should be substantiated with more data and analyses.

**Is the method likely to be of broad utility? Is any software component easy to install and use?**

RNA degradation is an important problem when working with clinical samples, especially when samples come from different sources.

**Is the paper of broad interest to others in the field, or of outstanding interest to a broad audience of biologists?**

**Yes**: Yes, see answer to previous question.

**Comments to author:**

The manuscript entitled "Normalization of generalized transcript degradation improves accuracy in RNA-seq analysis" by Xiong and colleagues reports on a novel data-analysis method to detect and correct transcript specific integrity issues. Through combined normalization/integrity correction, the authors claim higher accuracy of the results. With limited datasets explored (given the complexity of RNA degradation) and too few alternative normalization methods tested, the generality of the claim is not fully supported.

major comments:
1. The authors only studied 'naturally degraded' RNA, but did they also consider 'fragmented' RNA, e.g. from formalin fixed paraffin embedded tissues? Please note that there is a difference between degradation and fragmentation, in underlying cause, pattern and transcript specificity.
2. The authors claim that degradation typically happens at the 5' end. My understanding is that it can occur at 5', 3' and along the transcript. More importantly, can the proposed method handle different types of degradation?
3. Why did the authors select UQ, TIN, RUVr and RUGg as the 4 normalization methods to compare their method with? They should at least include a few more popular methods, such as the one implemented in DESeq and TMM. Suppose the selected 4 don't work well, then it's not too difficult to demonstrate better performance of DegNorm.
4. In line with the previous comment, in my opinion the authors failed to demonstrate that their method is substantially better than the best one tested.
5. One should be cautious with simulation data that is generated by the developer and tester of the method that will use the simulation data. There is inherent risk of built-in biases and using assumptions that work well for the developed method. Can the authors reduce my concern and discuss this in their manuscript?
6. Discussing a possible reduction in false positives is pointless without concomitant assessment of false negatives. In other words, sensitivity and specificity should be simultaneously assessed (and perhaps also other metrics such as true positive rate).
7. Figure 4: DegNorm (and TIN) appears to be "more effective to correct for degradation", but it still seems problematic. Is it good enough? Will readers/users not (falsely) assume that it is okay to use degraded RNA when they would adopt such a normalization method?
8. The authors did not explore very well to what extend RNA can be degraded and what fraction of samples can be degraded when doing differential gene expression analysis and still obtain accurate results.
9. The authors should also benchmark their method using real clinical samples with known differential genes or pathways.
10. Figure 6 and interpretation in text: Does this mean that DegNorm removes alternative splicing signals?
11. The authors suggest that their method could also prove useful beyond degraded RNA. They could demonstrate this by comparing e.g. 2 different polyA+ library prep kits on the same samples, or compare polyA+ vs. total RNA lib prep on the same samples.
12. I would also like to see that DegNorm works well on high quality RNA samples.
13. The DegNorm code or script should be made available.

minor comments:
1. The authors should mention that nowadays RNA seq methods exist that can handle degraded/fragmented RNA, such as capture sequencing and total RNA sequencing. These methods may not need the proposed method for correction/normalization.
2. There are many flavors of RNA sequencing, so the authors should be more specific. I assume the method has only been tested (and may only be relevant) for RNA captured using its polyadenylated tail (polyA+ RNA seq), in contrast to (rRNA depleted) total RNA seq, 3' end seq, hybrid capture seq, etc.
3. In line with previous comment, the method is only tested on bulk RNA seq, which is fine, but this limitation should be mentioned.
4. It would be helpful that the pages are numbered.
5. At various occasions, "et al" is written while it should be "et al."
6. Results > data sets: please describe the 'emulated degradation' procedure. Also, how representative is this emulation? There are many types of degradation (enzymatic, heat, high salt concentration, etc.) and each may have a different impact on RNA.
7. In addition to a pairwise correlation matrix of DI scores, it is also useful to show at least one representative scatterplot. I would also like to see a scatterplot of DI values of 2 technical replicates, to assess how similar DI values are.
8. Figure 6 does not show log fold change of 18.23 as mentioned in the text.
9. Caveat is needed that spike-ins are not per definition true negatives. If the same amount of spikes are added to samples with different RNA content, then spikes will turn out to be differential after normalization using the endogenous genes.
10. The authors should provide numbers with respect to the compute intensive nature of their algorithm.

**Reviewer 3**

**Are the methods appropriate to the aims of the study, are they well described, and are necessary controls included?**
More detail on how the normalization was incorporated into the DE analysis would be useful.

**Are the conclusions adequately supported by the data shown?**

Not entirely. See comments.

**Are sufficient details provided to allow replication and comparison with related analyses that may have been performed?**

Code is needed.

**Does the method perform better than existing methods (as demonstrated by direct comparison with available methods)?**

Yes

**Is the method likely to be of broad utility? Is any software component easy to install and use?**

It will be of broad utility. I did not see anything about software availability.

**Is the paper of broad interest to others in the field, or of outstanding interest to a broad audience of biologists?**

**Yes**: see attached

**Comments to author:**

The paper suggests a method for accounting for gene and sample-specific RNA degradation to normalize RNA-seq data.

The method proceeds by assuming that each gene has a characteristic coverage curve in the absence of RNA degradation and that the observed counts should essentially be this curve adjusted for sampling depth. The template for the characteristic coverage curve is derived from all the samples and the heuristics that coverage should be uniform and that observed counts should be below the depth adjusted

curve.

Constrained NMF is used to estimate both the characteristic coverage curve and the true coverage. The constraint ensures that the observed counts are below the curve.

The basic idea seems quite powerful. The authors show using both real model data and simulated data that the normalization can appropriately remove some degradation effects. However, for the PBMC-S01 and GBM-R6vsR4 the method did not remove the effects. More on this below.

Comments:

1) The authors acknowledge that DegNorm will remove the effects of alternative splicing for DE analysis. Differential splicing is a biological effect that is seldom discussed in the context of DE analysis. I would prefer that the authors consider how their method could be used to detect alternative splicing, rather than argue that it could remove the effect.

A common form of alternative splicing is the utilization of alternative stop codons. Can this be distinguished from RNA degradation?

2) How could degradation cause "truly differentially expressed" genes? I thought the whole point of the normalization was to avoid inferring "differential expression" when it was actually differential degradation.

p. 13 "RNA Degradation induces bias and thus may cause extra variance or even true differential expression if some transcripts are completely degraded."

I would not call this "true differential expression". I would say that no method can recover completely degraded transcripts and thus the inference of differential expression cannot be avoided.

p. 13 " For PBMC-S01 and GBM-R6vsR4 comparisons with known but mild mRNA degradation variation, the ECDF from different methods were all well above the diagonal line (except TIN for PBMC data), suggesting that degradation probably have caused true differentially expressed genes (Figs. 4f, g)."

These plots show a considerable deviation from the theoretical y=x line. It seems unlikely that degradation was so severe that this many transcripts were completely degraded. My conclusion would be that the method is not powerful enough to correct for severe degradation. Possibly this is due to the problem of trying to find the envelope curve when there is severe degradation.

3) How is does the DE analysis incorporate the normalization? In packages such as edgeR and DESeq2 we usually use an offset in the model so that the mean-to-variance relationship is not disturbed. Using adjusted counts is not correct for the negative binomial regression model.

It is not even clear that the usual negative binomial regression model (which posits the same dispersion for each gene) will be correct, as degradation will likely increase the dispersion.

4) I understand your justification for expecting the degraded counts to be below the curve, but due to Poisson (or negative binomial) variation in the counts this might be violated. Also, considering the type of variation, would it make more sense to use a weighted least squares or Bregman decomposition rather than least squares?

Minor comments:

p. 21 singular-value-decomposition I thought it was NMF. I see in the supplementary material that the SVD is used to compute the NMF, but I think that for clarity in the main body of the paper, where this detail is not explained, it is best to call it NMF.

p. 21 "prinounced" should be pronounced

Fig 5 caption "of in"

Fig. 5 caption: h is ECDF not CV

Supplementary material

Use -f(\lambda) so that you are always discussing convex functions.

p. 4 "fours"

Supp Fig 4. line 2 "estiamated" I do not understand what is in the boxplot. Why not use a scatterplot if you want to compare estimated to true? Or a boxplot of the difference or ratio?

**Authors Response**

We are very grateful for the insightful and helpful comments by all three reviewers. We first summarize the major changes we have made in this revision below: We have analyzed additional data sets per recommendation by one reviewer. In the previous version, all five data sets were from cell line samples under poly(A) selection (mRNA-seq). In this revision we have included two RNA-seq data sets from clinical samples. The first is the DLPFC data from both poly(A)+ and Ribo-Zero-seq protocols. We investigated whether DegNorm method also works effectively for degradation normalization for Ribo-Zero-seq data (ribosomal depletion protocol), or cross-protocol DE analysis. The second one is Breast Tumor mRNA-seq data of matched fresh frozen (FF) and Formalin-Fixed Paraffin-Embedded (FFPE) tissues. We investigated whether DegNorm can improve DE analysis that concerns highly fragmented FFPE samples. We removed the Zebrafish data from this version due to limit of space and for lack of ground truth to rigorously assess performance of different methods besides the spikein controls. The main figures order remains the same but with new contents added (results about zebrafish data deleted). We added one new figure, Supplementary Fig. 4. The order of subsequent figures are adjusted accordingly. All reviewers commented about the computing or software tool issue. While making this revision, we have completed a test-version of a comprehensive Python package that implements the DegNorm pipeline. This DegNorm package takes RNA-seq read alignment files (.bam files, single-end or paired-end) and a gene annotation file (.gtf) as input. It first calculates the coverage curves defined on the total transcript, i.e., each gene’s exons are concatenated. This functionality itself will be useful for visualizing the reads distribution for any gene of interest. To the best of our knowledge, there is no other package/tool available that has similar functionality (SAMTOOLS can calculate the read coverage in the genome, but the paired ends, when overlapping with each other (which frequently happens in RNA-seq), will double count the coverage score in the overlapping region. The core function of DegNorm is the degradation normalization procedure, and is surfaced to users as a command line interface (CLI) tool. It outputs degradation-normalized coverage curves, read counts and additional plots (heatmap, boxplot etc). The normalized read counts will be ready for DE and other downstream analysis. This package is available at https://nustatbioinfo.github.io/DegNorm/. Computing time: The computing time depends on several factors. (a) The level of RNA degradation in the samples. More degraded samples require more intense computation to determine the “baseline regions” of relatively non-degraded coverage. (b) The number of genes, and to a lesser extent, the number of samples, being considered. (c) The number of CPUs available for parallelization. (d) The number of NMF-OA iterations per approximation, and the number of DegNorm iterations. Decreasing either iteration count will trade accuracy for speed. For example, for the FF VS FFPE comparison (3 vs. 3, 11670 genes) presented in this revision, it took about 9 hours to complete the entire pipeline on a cluster node of Intel Xeon 2.80GHz CP with 22 parallelized threads. We are currently working on a version with MPI implementation that can parallelize computing load over separate compute nodes, which will greatly speed up the algorithm.

**Point-by-point responses to the reviewers’ comments:**

**Reviewer 1**

The authors proposed a novel normalization method for RNA-seq data that takes care of the degradation effect. Interestingly, their definition of degradation effect not only refers to the mRNA degradation, but also the alternative splicing, etc. This clever way bypasses the difficulty of deconvoluting the mRNA degradation effect and alternative splicing, which is virtually impossible, and gives a uniformed and easy-to-use method for normalizing individual genes. Figure 6 clearly demonstrates the very impressive performance of their method. The correction of degradation from the left panel to the right panel, especially considering the nonparametric shape of the mRNA degradation and the complicated alternative splicing pattern, seems almost impossible, but the proposed method works almost perfectly.

The algorithm they propose is solid and effective. The authors carefully pick up four real datasets, each with its own characteristics, to demonstrate different aspects of the performance of their method. Their method consistently outperforms existing methods on all four datasets. They also carefully studied their method and compared with others on simulation data. The pipeline, if made publicly available and user friendly, will be very widely used. I expect high impact of their method.

The paper is very well written. All the real datasets have been thoroughly analyzed and the discussion about the comparison is fair and sound. I have no major concerns, and all my comments/suggestions below should be considered minor. In the description of methods, the authors consider all read counts along "expressed eons in the sequential order". How about junction reads (That is, reads that connecting exons)? Did the authors just connect all exons into a large transcript (then how about alternative splicing?) or did the authors just ignore all junction reads? Including junction reads uses more information and hence may increase power, but it will also complicate things. Whatever way the author was using should be fine and does not need to be changed, but it is a good idea to describe it a bit more clearly.

*We defined the total transcript by concatenating all annotated exons for a given gene. If there are junction reads, the reads will be split between respective junctions (not necessary consecutive junctions) in the calculation of read coverage score. They are NOT ignored. It’s possible that different samples may express different splicing forms. The defined total transcript will allow visualization of such alternatively spliced exons, and facilitate the comparison of coverage curves between samples in the DegNorm algorithm. We have clarified this on page 10 of Supplementary materials in the revision.*

Since Finotello et al (21) proposes a simple method for reducing degradation effect, is it possible to compare the proposed method with theirs?

*The maxcount in Finotello et al (21) was defined as the maximum number of reads that cover any one position within the same exon. We did consider this approach, but we found there are several issues. First of all, this method was proposed for exon-based DE comparison, instead of whole-gene level based comparison. In other words, it’s not directly comparable in the gene level DE analysis presented in this paper. Secondly, only counting the maximum number of reads that cover one position within the same exon leads to loss of the read counts, which consequently undermine the power of the DE analysis (in the count based on methods such as Negative Binomial model implemented in edgeR and Deseq). Thirdly, in many cases, the entire exon region could be subject to degradation, maxcount in this exon does not provide necessary correction (e.g. Gene ACTN4 in Figure 1d for the GBM data).*

The authors do not clearly describe how they did DE analysis after normalizing the data using DegNorm. It seems that they used edgeR (I learned this from the legend of Figure 8)?

*Thanks for pointing out this. We have clarified it in the last paragraph on page 13 “We normalized the raw read count by dividing it by 1- DI score for each gene within each sample. The adjusted read counts (rounded) were input into edgeR package [25] for DE analysis.”*

There is a typo on line 34 of the page before "Figure Legends": "prinounced" should be "pronounced". *Thanks for pointing out this. We have corrected this error in the revision.*

**Reviewer 2**

The manuscript entitled "Normalization of generalized transcript degradation improves accuracy in RNA-seq analysis" by Xiong and colleagues reports on a novel dataanalysis method to detect and correct transcript specific integrity issues. Through combined normalization/integrity correction, the authors claim higher accuracy of the results. With limited datasets explored (given the complexity of RNA degradation) and too few alternative normalization methods tested, the generality of the claim is not fully supported.

major comments:

The authors only studied 'naturally degraded' RNA, but did they also consider 'fragmented' RNA, e.g. from formalin fixed paraffin embedded tissues? Please note that there is a difference between degradation and fragmentation, in underlying cause, pattern and transcript specificity.

*Thanks to the reviewer for pointing us to such data. We have included two clinical data sets in this revision, one to assess the model performance in Ribo-Zeroseq and one to compare FF vs. FFPE data in DE analysis.*

The authors claim that degradation typically happens at the 5' end. My understanding is that it can occur at 5', 3' and along the transcript. More importantly, can the proposed method handle different types of degradation?

*We agreed with the review that degradation has multiple pathways, and can happen in any region within a transcript, not only in the 5’ end. This was stated (line 19, Page 4, Ref #16) in the original version. But we did say “The degradation typically happens in the 5’ end of transcript” in the manuscript because all data sets considered in previous version was mRNA-seq data. We have deleted this sentence and rephrased it on page 4.*

Why did the authors select UQ, TIN, RUVr and RUGg as the 4 normalization methods to compare their method with? They should at least include a few more popular methods, such as the one implemented in DESeq and TMM. Suppose the selected 4 don't work well, then it's not too difficult to demonstrate better performance of DegNorm.

*We compared 7 different methods rather than four, including UQ, TIN, RUVr, RUVg, TMM, RLE and TC. These methods are all commonly used in RNA-seq normalization. Methods including TMM, RLE (implemented in DESeq2 as the Review indicated) and TC (total counts) didn’t show pronounced difference compared to UQ method in the data we analyzed. So we decided to present these three methods in the Supplementary Fig. 3 for visualization convenience (remains as Supplementary Figs. 3 in this revision). The description of these methods were in the end of subsection “DegNorm improves accuracy in gene expression analysis” in the original version. We have now moved it to the beginning of this Subsection.*

In line with the previous comment, in my opinion the authors failed to demonstrate that their method is substantially better than the best one tested.

*First of all, as we replied in comment #3 above, we compared DegNorm with 7 different normalized methods, not only 3. In practice, we desire a method that shows consistently better performance than other methods in every situation. Such a method however may not exist. A method that achieves overall robust and relatively better performance is preferred. We are not sure, which “best one tested” the reviewer was referring to in this comment. It appears clear to us that none of the other methods got consistently better or even close overall performance in the data sets we considered here (although may in individual cases).*

The DegNorm is designed for correction of degradation bias, particularly when degradation pattern differs between different conditions or samples. The motivation is that most of RNA-seq samples come with some degradation, more or less. A sample with RIN >=7 has been generally regarded as good quality samples in practice (personal communication with NU sequencing core Director Dr. Xinkun Wang). We showed not only in the cell line data (PBMC, GBM, SEQC-AB), but also in clinical samples (DLPFC, Breast Tumor), DegNorm convincingly shows significant improvement over other methods under comparison regardless whether there is known degradation difference or not. This was evidenced by the ECDF curves of p-value that are consistently closer to the diagonal line in the data where there is no true differential expression (PBMC, GBM, DLPFC mRNA-seq, DLPFC Ribo-Zero-seq and FF vs FFPE comparisons) and high ROC curves in SEQC-AB data.

One should be cautious with simulation data that is generated by the developer and tester of the method that will use the simulation data. There is inherent risk of built-in biases and using assumptions that work well for the developed method. Can the authors reduce my concern and discuss this in their manuscript?

*We agree with reviewer on this. Our conclusion is more weighted on the results from the real data rather than simulation. In particular, following the reviewer’s suggestion, we have analyzed Ribo-Zero-seq data, and clinical FF vs FFPE data in this revision. These RNA-seq data have different mechanisms of degradation from the mRNA-seq data. All results suggest that DegNorm achieves better effectiveness in reduce degradation bias in the DE analysis.*

The simulation of data is always done under some assumptions. The real data is always more complex than the simulations. But we wish the simulation of the degradation can mimic the true degradation, and the results can provide insights into what are observed in the real data. For example, in this paper, we used the cumulative distribution function to simulate the degradation pattern (SM_Figure 1 in Supplementary Methods). This is one way to simulate the degradation what occurs in the 5’ end from commonly used poly(A) selection protocol. Once these data have been simulated, we pretended to know nothing about the assumptions of model that generated the degradation. DegNorm is purely data-driven, and it does not make any assumption regarding what model was used to generate the degradation. For example, if 3’ degradation or internal degradation is the main cause, DegNorm can also be readily applied. The simulation study did provide insights to what we’ve seen in the real data regarding degradation pattern, CV, ECDF of p-values and ROC curves. For example, Simulation III presented a case where degradation pattern is clustered as observed in the real data SEQC-AB, which may explain why DegNorm performs better in the SEQC-AB data.

Discussing a possible reduction in false positives is pointless without concomitant assessment of false negatives. In other words, sensitivity and specificity should be simultaneously assessed (and perhaps also other metrics such as true positive rate).

*We totally agree with the Reviewer on this. Sensitivity and specificity are two important measures to assess the performance of a method. Note that specificity = 1- false positive rate, and sensitivity = true positive rate. That’s why we did present the ROC curves for SEQC-AB data and for all simulation data where we know the ground truth. The X-axis of ROC is 1-specificity (false positive rate) and Y-axis is the sensitivity (true positive rate). This evaluation can only been done when the ground truth is known or there exists a large set of validated genes*

*For SEQC-AA, PBMC, GBM, DLPFC mRNA-seq, DLPFC Ribo-Zero-seq and Breast tumor FF vs FFPE data, all hypotheses were originally negative. In theory, we don’t have any false negative from this perspective. Thus all positives can be regarded as false positives. That’s why we discussed mostly about reducing false positives in these data sets.*

Figure 4: DegNorm (and TIN) appears to be "more effective to correct for degradation", but it still seems problematic. Is it good enough? Will readers/users not (falsely) assume that it is okay to use degraded RNA when they would adopt such a normalization method?

*Certainly that’s not good enough. All normalization methods are post-hoc approaches that try to reduce systematic biases from different causes, so for DegNorm. If degradation has caused dramatic change of transcript abundance level, no methods can completely recover it. We have discussed it on page 21 and stated that data of high quality is always desired.*

The authors did not explore very well to what extend RNA can be degraded and what fraction of samples can be degraded when doing differential gene expression analysis and still obtain accurate results.

*It’s hard to give a simple answer to this question. First of all, the main challenge is that degradation not only varies between samples, but also between genes. The RIN number only provides sample-wise overall summary statistic for average degradation in the given sample (evaluated from ribosomal RNA species). Secondly, From the GBM R10 vs R4, R6 vs R4 comparisons, we learned that not only the average degradation within samples matters, but also the relative degradation level and homogeneity of degradation pattern between samples. In the following we conceive three naïve examples, by which we hope to clarify this point.*

*In the second situation, again two samples each start with 100 copies of transcript for the same gene. The transcripts in first sample are complete without degradation, while transcripts in the second sample have severe degradation. The severe degradation causes complete degradation of some transcripts in the second sample, and only 75 copies of partially degraded transcripts remain in the second sample upon RNA-seq. In this situation, DegNorm can utilize the partial transcript information based on coverage curve and recover the lost read count from the 75 copies in the second sample, but not for the completely disappeared 25 copies. In this case, DegNorm can reduce the statistical significance (in terms of p-value), but it may not completely cure this problem. This is a similar situation in the RIN10 vs RIN4 example. The RIN 4 examples were severely degraded. The p-value ECDF was pulled down under DegNorm (and TIN) compared to other methods, but still way above the diagonal line.*

*In the third situation, we had two samples each starting with 100 copies of transcript for the same gene. Both samples have severe but comparable degradation, each having around 10 - 25 copies completely degraded. The remaining partially degraded transcripts help to estimate the coverage curve to correct for the read loss in the reaming transcripts. In this case, the total number of remaining copies may be still close in the two samples. The RIN6 vs RIN4 comparison in Figure 4 presents a similar case as this setting, where DegNorm can better reduce false positives compared to other methods as shown in the ECDF of p-value plot.*

*So the conclusion is that not only the absolute degradation level of each sample matters, but also the relative degradation level and homogeneity of degradation pattern.*

The authors should also benchmark their method using real clinical samples with known differential genes or pathways.

*Following reviewer’s recommendation, we have included two clinical data sets in this revision, one from dorsolateral prefrontal cortex (DLPFC) tissue of five brains under both mRNA-seq and Ribo-Zero-seq protocols (ref #22) and the other Breast Tumor samples under FF and FFPE methods (ref #29). The DE analysis we considered were groups of samples that had no true differential expression except degradation time (DLPFC), but under different protocol or different sampling preparation methods. In this case, we can assess the false positive rate by examining the p-value curve. Clearly there are many clinical RNA-seq data sets available that had biological difference between conditions. As the reviewer commented earlier, we need to evaluate both false positive and false negative rate. For being an ideal data set for the assessment of a method, only knowing a handful genes that have differential expression is not adequate. We desire to have one that has large-scale PCR validation for differentially expressed genes for assessment of sensitivity and specificity. SEQC-AB data is one such ideal data set. From SEQC-AB data, we can see that if without the PCR validation results, it’s extremely difficult to judge which method works relatively better. For example, the ECDF curves of DegNorm and TIN were both lower than other methods in Figure 5c. It could be understood as that DegNorm and TIN tend to reduce more false positives (good) or that DegNorm and TIN tend to induce more false negatives (being less powerful, bad) or a mixed of the two. The ROC curves based on PCR results elucidated that the much lower ECDF curve of TIN method is due to greatly inflated false negative rate. In contrast, the ROC curve of DegNorm suggests that the lower ECDF of p-value in DegNorm is an indication of more reduced false positive rate without sacrificing the power.*

*We have looked into some additional data sets that reviewer has pointed out to us, and were not able to find a suitable clinical data sets that have similar large-scale validations as SEQC-AB data.*

Figure 6 and interpretation in text: Does this mean that DegNorm removes alternative splicing signals? *We assume the Reviewer referred to the NDUFV3 gene in Figure 6e,f. The B samples of NDUFV3 did show much depleted coverage in the second exon region, likely due to alternatively splicing. But one could also argue that the lower read counts in the second exon region of B samples were caused by degradation since degradation can occur in any region of the transcript. Yes, DegNorm did fill in the gap in the B samples in the second Exon region to level up the coverage curve, and use it to calculate normalized read count. DegNorm is designed for improving differential expression analysis (i.e. statistical test for the relative abundance of transcript copies), not for detecting alternative splicing. In practice degradation and alternative splicing are often confounded and could be difficult to distinguish. In the DegNorm package we developed, both the coverage curves before and after normalization will be available. The raw coverage curves before normalization could be used for visual detection of possible alternative splicing.*

The authors suggest that their method could also prove useful beyond degraded RNA. They could demonstrate this by comparing e.g. 2 different polyA+ library prep kits on the same samples, or compare polyA+ vs. total RNA lib prep on the same samples.

*We thank Reviewer for this great suggestion. Our lab is a dry lab and unfortunately we cannot perform such experiments. But we found some public data (Ref #22), and did comparison between RNA-seq data under poly(A)+ vs. Ribo-Zeroseq. The conclusion is that none of the methods considered can generate satisfactory results in such cross-platform comparison (Supplementary Fig. 4 B). Our explanation is that the RNA-seq reads sequenced under the two protocols probably don’t represent the same population (i.e. the parental RNA fragments in the sample). It is not recommendable to do DE expression between such data sets.*

I would also like to see that DegNorm works well on high quality RNA samples.

*SEQC-AA and SEQC-AB were created by the international RNA-seq quality control consortium for quality control purpose, and they are considered as high quality data (but we were not able to find out the RIN number attached to each sample). We also communicated with the Northwestern Sequencing Core Director Xinkun Wang about the RNA-seq sample quality issues. It seems to be quite common to have RIN is around 7 or even below it. As RIN number was measure based on 28S to 18S rRNA ratio, it may represent the average RNA sample quality, but the degradation may still vary between genes. For example, longer ones may have worse degradation than shorter ones. Therefore we think degradation maintain as an issue in samples of good quality.*

The DegNorm code or script should be made available.

*We have made R codes and a python package available at* [*https://nustatbioinfo.github.io/DegNorm/*](https://nustatbioinfo.github.io/DegNorm/)*.*

minor comments:

The authors should mention that nowadays RNA seq methods exist that can handle degraded/ fragmented RNA, such as capture sequencing and total RNA sequencing. These methods may not need the proposed method for correction/normalization.

*In addition to inclusion of clinical samples of ribo-zero-seq, FF and FFPE, we have added the limitation of the method the discussion section.*

There are many flavors of RNA sequencing, so the authors should be more specific. I assume the method has only been tested (and may only be relevant) for RNA captured using its polyadenylated tail (polyA+ RNA seq), in contrast to (rRNA depleted) total RNA seq, 3' end seq, hybrid capture seq, etc.

*Reply: This paper was originally focused on the mainstream protocol on RNA-seq, or the mRNA-seq, Under reviewer’s suggestion, we also tested on the Ribo-zero-seq data, it seems to work as well as in the mRNA-seq data, and even for FF vs FFPE comparison (under same mRNA-seq protocol). We haven’t test this method on other types of RNA-seq like 3’seq. This limitation has been discussed in the discussion section.*

In line with previous comment, the method is only tested on bulk RNA seq, which is fine, but this limitation should be mentioned.

*Yes. This limitation has been discussed in the discussion section.*

It would be helpful that the pages are numbered.

*Yes, in the revision pages has been numbered.*

At various occasions, "et al" is written while it should be "et al."

*Thanks for pointing out this. We have made corrections throughout.*

Results > data sets: please describe the 'emulated degradation' procedure. Also, how representative is this emulation? There are many types of degradation (enzymatic, heat, high salt concentration, etc.) and each may have a different impact on RNA.

*We have added brief description into the text. Detailed description can be found in original paper cited in this manuscript.*

In addition to a pairwise correlation matrix of DI scores, it is also useful to show at least one representative scatterplot. I would also like to see a scatterplot of DI values of 2 technical replicates, to assess how similar DI values are.

*We have added three scatter plots in Supplementary Fig. 1 to illustrate the DI scores between samples of the same RIN or different RINs.*

Figure 6 does not show log fold change of 18.23 as mentioned in the text.

*This is a great catch. It seems that RT-PCR results and RNA-seq may have large discrepancy in some cases although the former has been widely used as a validation tool. We have replaced this one by a different example in this revision.*

Caveat is needed that spike-ins are not per definition true negatives. If the same amount of spikes are added to samples with different RNA content, then spikes will turn out to be differential after normalization using the endogenous genes.

*The ERCC controls in practice are usually spiked into RNA samples (or microarray) at identical concentration for each given transcript species (https://www.nature.com/articles/nmeth1005-731). They are generally regarded as negative controls. By design, people hope use ERCC controls to normalize the RNAseq read counts of other genes, not the reverse way, i.e., normalizing ERCC by other genes in the same sample. In the latter case, clearly inappropriate choice of exdogenous genes may result in false positives of ERCC in differential expression analysis (we agree with the reviewer on this).*

*In practice, however it has been found that using ERCC controls for normalization is not satisfactory (ref #8 and others). The motivation to present the coverage curve of ERCC-00019 in zebrafish data in Figure 1C (deleted in this revision) was to show the different shapes of coverage curves, which could contribute to the unsatisfactory performance of using ERCC controls for normalization in RNA-seq. As ERCC controls are regarded as true negatives and other sources of biases may increase the relative variability, they are frequently used to assess the performance of normalization methods by examining the coefficients of variance (CV). Figure 5G in the original version was intended to show the reduced CV under DegNorm compared to the UQ method.*

*As the zebrafish data had biological difference between the conditions, but had no validation data, it does not provide convincing evidence of false and true positive rate. Due to the limit of space, we dropped it from the current version.*

The authors should provide numbers with respect to the compute intensive nature of their algorithm.

*We have commented in the discussion section of the main text.*

**Reviewer 3**

The authors acknowledge that DegNorm will remove the effects of alternative splicing for DE analysis. Differential splicing is a biological effect that is seldom discussed in the context of DE analysis. I would prefer that the authors consider how their method could be used to detect alternative splicing, rather than argue that it could remove the effect. A common form of alternative splicing is the utilization of alternative stop codons. Can this be distinguished from RNA degradation?

*Indeed that alternative splicing is rarely discussed in DE, simply because statistical tests for differential expression using read counts from RNA-seq implemented in tools like edgeR and DEseq are not designed to detect alternative splicing. In gene-level analysis, DE is to test the equivalence of transcript relative abundance of a gene between two conditions through the tag counts, and they are not informative for testing alternative splicing. The relative abundance, sequencing depth, transcript degradation and alternative splicing are four factors that all affect the read counts for a given gene. In particular transcript degradation and alternative splicing are often confounded. If one sample has dramatically reduced read count/coverage score on one given exon than the other sample, it can be understood either as degradation or possible alternative splicing. They are often hard to distinguish or separate from each other. In both cases, for testing the relative abundance of copy number of transcripts using the tag counts, we argued that the under counts of tags due to degradation or depleted exon usage need to be compensated. The degradation normalization is designed for this purpose.*

*Identification of the alternative splicing forms is extremely challenging when degradation is present. We cannot simply test the read counts exon by exon because of the differential read counts could be due to degradation. Stop codon or start codon could help in finding different splicing form with different initial or terminating exon. But alternatively splicing frequently may involve the internal exons that don’t involve start or stop codon, for example in the SEQC-AB-NDUFV3 example in Figure 6e,f (if we assume it is truly alternative splicing). One can argue the lower coverage score in the second exon region of B samples are due to alternative splicing, but one can also argue it’s due to degradation since degradation may occur at any region of the transcript.*

*In the software package DegNorm we developed, we provide functions to visualize the coverage curves before and after degradation normalization as shown in Figure 6. The visualization of the coverage curves could provide clues for manual identification of alternative splicing.*

How could degradation cause "truly differentially expressed" genes? I thought the whole point of the normalization was to avoid inferring "differential expression" when it was actually differential degradation. p. 13 "RNA Degradation induces bias and thus may cause extra variance or even true differential expression if some transcripts are completely degraded." I would not call this "true differential expression". I would say that no method can recover completely degraded transcripts and thus the inference of differential expression cannot be avoided. p. 13 " For PBMC-S01 and GBM-R6vsR4 comparisons with known but mild mRNA degradation variation, the ECDF from different methods were all well above the diagonal line (except TIN for PBMC data), suggesting that degradation probably have caused true differentially expressed genes (Figs. 4f, g)." These plots show a considerable deviation from the theoretical y=x line. It seems unlikely that degradation was so severe that this many transcripts were completely degraded. My conclusion would be that the method is not powerful enough to correct for severe degradation. Possibly this is due to the problem of trying to find the envelope curve when there is severe degradation.

*Sorry for the confusion. We agree the wording was confusing. For SEQC-AA, PBMC-S01, GBM, DLPFC and Breast Tumor, samples originated from groups that had no DE, and thus all hypotheses were originally negative. In theory, we don’t have any false negative from this perspective. Thus all positives can be regarded as false positives. In the meanwhile, severe degradation may change the abundance of transcript of some genes when they are up for sequencing. We have rephrased this sentence to " RNA Degradation induces bias and thus may cause extra variance. Severe degradation may even result in difference of transcript abundance for some genes when they are prepared for RNA-seq." on page 13. For GBM data, the RIN 10,6,4 samples were all normalized together. We believe the envelop function were well estimated because of the presence of RIN 10 samples (e.g., Fig. 6a,b). Thus we interpret that substantially off-diagonal line of p-value curve in GBM-R6vsR4 (same as R10vsR4) is likely due to the difference of transcript abundance caused by loss of transcript from complete degradation of some genes in the samples.*

*Similarly, for the PBMC-S01 data, we also have the sample at 0 hour, which is presumably to be more complete than other times points. In fact Figure 3d suggests that degradation only slightly increase from 0, 12, to 24 hours, while substantially increased at 48 hours. Thus to estimate the envelop may be not an issue, but rather the elevated degradation in 24 and 48 was likely the cause of off-diagonal line of pvalue curve in the 0+12 vs 24+48 comparison.*

*Perhaps the “mild” word is inappropriate, we have replaced it by “modest” in the revision the two comparisons in GBM-R6vsR4 and PBMC.*

*DegNorm is more effective when transcripts of a gene are partially degraded, but the copy number remains about the same. In this case the lost portion of read counts can be compensated by estimating the envelope function using existing transcript information from all samples. If a substantially proportion of the transcripts are completely degraded, DegNorm will not be able to completely remove the degradation effect, probably no methods could since the degradation severity is gene specific.*

How is does the DE analysis incorporate the normalization? In packages such as edgeR and DESeq2 we usually use an offset in the model so that the mean-tovariance relationship is not disturbed. Using adjusted counts is not correct for the negative binomial regression model. It is not even clear that the usual negative binomial regression model (which posits the same dispersion for each gene) will be correct, as degradation will likely increase the dispersion.

*We are sorry that we did not clearly state it in the main text in the original version. We have added on sentence on page 13 “We normalized the raw read count by dividing it by 1 minus the DI score for each gene within each sample. The adjusted read counts (rounded) were input into edgeR package [25] for DE analysis” . This was again re-stated in the Methods section, page 31.*

*The Reviewer raised a great question about how to configure the normalization factor into the negative binomial model. We understand that the offset estimated by edgeR and DESeq2 plays the role of global normalization scaling factor, but they are operating on the NB regression parameters, rather than the read counts themselves such that the read counts can maintain integer values. However with the degradation bias, what read counts really follow the NB regression model first becomes a question. The DegNorm normalized count is given by X ̃_ij=X_ij/(1-ρ_ij) where X_ij is the raw read count for gene i in sample j, and ρ_ij is the estimated degradation index score at convergence. We applied edgeR in the DE analysis by inputting X ̃_ij (rounded to integer) as read counts throughout this paper. Our assumption is that the latent true count (i.e., count without degradation) has negative binomial distribution. X ̃_ij provides an estimator of the latent true count. edgeR will still estimate a sequence depth normalizing factor depending on which method was used (UQ, TC, TMM etc). From this perspective, we think the negative binomial is still valid. From practical point of view, probably it does not matter. For example, many other normalization methods including RUVg, RUVr, TIN, quantile normalization, SVA etc all operate on the read counts and have to round the normalized counts before they are plugged in for DE analysis.*

I understand your justification for expecting the degraded counts to be below the curve, but due to Poisson (or negative binomial) variation in the counts this might be violated. Also, considering the type of variation, would it make more sense to use a weighted least squares or Bregman decomposition rather than least squares?

*The Reviewer is exactly right. The Poisson or NB noise was not taken into consideration in the DegNorm pipeline. The good side of this solution is that we get elegant and simple solution for possible degradation bias correction, but paying a price of over-approximation of degradation when the coverage curve fluctuates due to noisy, particularly when the signal is low (Figure 2, S3 and S4, Figure 7e). We have repeatedly discussed this issue in the text. We have thought to define the envelop function in terms of expectation, but we haven’t been able to figure out a better and simple solution yet. As different samples may have different abundance level (reflected by the scale parameter K in our algorithm), and within the same sample, different regions of the transcript may have different degradation, we cannot take a point-by point approach. In addition, the coverage scores at neighboring positions are not independent because of the moving average nature how the coverage scores were calculated.*

*We are not familiar with the Bregman decomposition. But we are grateful that the Reviewer pointing us to this direction. We would like to explore it further in the future.*

Minor comments:

p. 21 singular-value-decomposition I thought it was NMF. I see in the supplementary material that the SVD is used to compute the NMF, but I think that for clarity in the main body of the paper, where this detail is not explained, it is best to call it NMF.

*Thanks for this great suggestion. We have changed it in the main text.*

p. 21 "prinounced" should be pronounced

*corrected.*

Fig 5 caption "of in"

*corrected*

Fig. 5 caption: h is ECDF not CV

*We are confused. Fig 5h is indeed the CV plot of DegNorm vs UQ, not ECDF. In this revision, we have removed the zebra fish data and replaced by two new data sets.*

Supplementary material Use -f(\lambda) so that you are always discussing convex functions.

*We feel it’s natural to keep f(\lambda) as is till equation (3) on page 7. When starting to talk about optimization of f(\lambda) after (3), we followed the reviewer’s suggestion and changed to –f(\lambda), so it’s consistent with the definition of subgradient on page 7. Also we changed the notation f(x) to g(x) in the definition of subgradient on page 7 to avoid confusion with f(\lambda).*

p. 4 "fours"

*Corrected. Thanks.*

Supp Fig 4. line 2 "estiamated" I do not understand what is in the boxplot. Why not use a scatterplot if you want to compare estimated to true? Or a boxplot of the difference or ratio?

*Typo corrected.*

*The boxplots are for the true degradation scores vs estimated degradation scores for each simulation. The main purpose of Supp. Fig. 5 (a-f) (original version SF4 (a-f)) was to show whether DegNorm can recover to big picture of degradation of each sample. This is kind of providing a marginal distribution of degradation scores. For example, in Simulation IV, we see elevated degradation in the B samples as shown in the ground truth. If we just plotted the scatter plot of true or estimate DI scores, we will need to plot 8 scatter plots for each simulation (or we can merge to one), in which case we will not be able to easily see the different of degradation difference between samples as shown in the box plot.*

*Supp. Fig. 5 (g-l) were to show whether DegNorm can recover the degradation pattern between samples/conditions. The pairwise correlation was calculated based on the DI scores of all genes. For example, In Supp Fig. 5 k and l, we showed that DegNorm can recover the clustered degradation pattern within the same condition (similar to the ground truth). This echoes what was observed in the SEQC-AB data, by which we hope provide some insights into why DegNorm could improve the DE analysis in such situations.*

*Rather than presenting scatter plot of DI scores, we think it’s more informative to plot normalized read counts vs true counts (Figs.7e, 8c Supp Fig. 6c,g), as they not only can show how good the estimated DI scores are in recovering the true counts (or equivalently the true DI scores), but also when.*

*For these considerations, we have kept the Supp Fig 5 as is.*

**Second round of review**

**Reviewer 2**

The authors went to a great length in addressing my and the other referees’ comments. However, there are still some outstanding issues. 

In reply to my comment “In line with the previous comment, in my opinion the authors failed to demonstrate that their method is substantially better than the best one tested.”, the authors state that “it appears clear to us…’. I would appreciate if the authors would objectively quantify this statement. 

In reply to “Discussing a possible reduction in false positives is pointless without concomitant assessment of false negatives. In other words, sensitivity and specificity should be simultaneously assessed (and perhaps also other metrics such as true positive rate).”, the authors state that “all hypotheses were originally negative” for the data sets mentioned. I want to point out that the null hypothesis is that there is no effect/change/difference. I would not say that the hypotheses are negatives. 

I’m not completely satisfied with the authors’ reply to “The authors did not explore very well to what extend RNA can be degraded and what fraction of samples can be degraded when doing differential gene expression analysis and still obtain accurate results.” Using simulations or subsampling data/samples (of varying qualities) from real data, the authors could at least attempt to assess impact of extent of RNA degradation and fraction of samples degraded. 

The same holds true for the reply to “The authors should also benchmark their method using real clinical samples with known differential genes or pathways.” Only assessing false negatives is not good enough. As proposed originally, I invite the authors to also include data from (partially) degraded/fragmented RNA samples with prior knowledge on differential pathways or genes. For sure, these exist. One could look for studies in which a transcription factor was silenced or upregulated and combined with known info on transcriptional target genes or regulated pathways, the authors could point out that their method is as good or better to recapitulate what is known from a transcriptional perspective. Other examples are the use of targeted drugs with well understood transcriptional effects; or comparing different cell types, organs, cell cycle stages, gender, healthy vs. diseases samples, etc. 

I’m satisfied with the reply to “The authors suggest that their method could also prove useful beyond degraded RNA. They could demonstrate this by comparing e.g. 2 different polyA+ library prep kits on the same samples, or compare polyA+ vs. total RNA lib prep on the same samples.”, however, I would like the authors commenting on their findings in the manuscript.

**Reviewer 3**

p. 10 "and a pairwise correlation matrix of DI scores between samples (Supplementary Figs. 1a-f)." 

Correlation would show that samples have a systematic gene-wise degradation regardless of overall quality of the degradation. I think given the argument that degradation can vary by gene, Euclidean distance makes more sense.

**Authors Response**

*We are very grateful for the continuous help/comments from the Editor and Reviewer. In the following we first summarize the changes, and then provide a point-by-point reply to Reviewer’s comments.*

*Minor changes:*

*We found some inconsistency of data processing for different data sets. In this revision, we have made corrections and made sure all data have been processed and analyzed exactly as described in the text. Accordingly, we made minor changes to a few figures including Figure 4a,e,f,k,l, sf4(a). Such changes do not change the conclusion of this paper.*

*Major changes:*

*Based on Review 2’s comments, we have provided quantitative measure of performance of different methods. The results are presented in numbers and figures to be described below. Additional tables and figured are added (Supplemental Table 1-3 and Figure 5a, Figure 10). In addition, we have included a new clinical data to illustrate different methods. Major changes of text are highlighted in blue color in the text.*

**Point-by-point responses to the reviewers’ comments:**

The authors went to a great length in addressing my and the other referees’ comments. However, there are still some outstanding issues.

1.In reply to my comment “In line with the previous comment, in my opinion the authors failed to demonstrate that their method is substantially better than the best one tested.”, the authors state that “it appears clear to us…’. I would appreciate if the authors would objectively quantify this statement.

*We have included numerical quantification and graphical comparison as follows:*

*For the six data sets that genes had not true differential expression, we compared false positive rate between different methods at q-value=0.05. The FDR of different methods over DegNorm was plotted in Figure 5a and Tabulated in Supplementary Table 1. The results are discussed in a new paragraph on bottom of page 15. Compared to DegNorm, UQ and RUVr increased the FDR by a factor ranging from 1 - 3.7 and 1.4 - 36.8 respectively. Although TIN beat DegNorm in FDR in three out the six, TIN method was demonstrated to have poor power in SEQC-AB data, four simulation studies and the new GBM AMPK knockout data. It’s not recommendable.*

*For SEQC-AB data, controlling FPR = 0.05 (specificity = 0.95), the larger PCR set suggested a 78.1% true positive rate (sensitivity) for DegNorm in comparison to 73.7%, 67.3% and 48.3% for UQ, RUVr and TIN methods respectively.*

*For the simulation studies, we compared the sensitivity (TPR) by controlling FPR = 0.05. The results are presented in Supplementary Table 3 and Figure 10. Simulation I does not contain any degradation bias or any other bias. Although DegNorm is not specifically designed for this setting, DegNorm had a performance very close to the latent (latent meaning using true read counts in the DE analysis). DegNorm had a decay of 0.6% in sensitivity compared to the latent at FPR=0.05 (UQ results are identical to latent as UQ perfectly normalize the sequencing depth when there is no any other bias, Fig. 8c). In contrast, RUVr and TIN resulted in a decay of 1.1% and 8.6% respectively compared to the latent. This was discussed on page 20.*

*In Simulation II-IV, DegNorm improved over all other three methods to different extents (results presented in Figure 10 and Supplemental Table 3). In particular for Simulation III, at FPR = 0.05 threshold, DegNorm improved sensitivity by a factor of 1.28, 1.30 and 2.01 over UQ, RUVr and TIN methods respectively (Fig. 10). These results were discussed in the last paragraph of page 21 and top paragraph of 22.*

*In summary, all the figures plus additional quantifications strongly support that DegNorm substantially improves over other methods under comparison in DE analysis.*

2.In reply to “Discussing a possible reduction in false positives is pointless without concomitant assessment of false negatives. In other words, sensitivity and specificity should be simultaneously assessed (and perhaps also other metrics such as true positive rate).”, the authors state that “all hypotheses were originally negative” for the data sets mentioned. I want to point out that the null hypothesis is that there is no effect/change/difference. I would not say that the hypotheses are negatives.

*By “all hypotheses were originally negative”, we meant that all null hypotheses were true. Although the wording might be a bit loose, we believe the meaning was clear in the context of the reply letter. We have carefully checked the main text and supplementary throughout, we didn’t find similar loose wording.*

3.I’m not completely satisfied with the authors’ reply to “The authors did not explore very well to what extend RNA can be degraded and what fraction of samples can be degraded when doing differential gene expression analysis and still obtain accurate results.” Using simulations or subsampling data/samples (of varying qualities) from real data, the authors could at least attempt to assess impact of extent of RNA degradation and fraction of samples degraded.

*In the reply letter of revision 1, we tried our best to explain why it’s difficult or even impossible to draw a conclusion regarding what extent or what fraction of samples can be degraded to get accurate results. We regret that it didn’t help. We wish the reviewer could help by pointing out why our explanations in the letter didn’t make sense or whether there was anything the he/she disagreed about. We would like to resummarize our points as follows.*

*The reviewer didn’t elaborate what he meant by "obtain accurate results". In DE analysis, suppose there are m0 true nulls, and m1 true alternatives (in total, m0+m1 genes). One could defined “accuracy” as the total number of corrected claimed genes, i.e. (#true positives +# true negatives)/(m0+m1). A more commonly used approach is to use the ROC curves. One could define “accuracy” as the sensitivity (TPR) at different FPR (or specificity) levels. Clearly the accuracy defined in either way not only depends on the degradation, but also depends on the sample size and effect size of testing. For example, even using the latent counts in Simulation I-IV, we cannot get a close to 100% sensitivity unless FPR threshold approaches 0.5 or above (Figure 9 km).*

*Now suppose we only focus on degradation bias, and controlling sample size, effect size and m0 and m1 all at about same setting. This question still cannot be answered easily for the following reasons:*

*First, unlike RIN numbers (one sample one RIN number), degradation score in our estimation (and in reality) is gene specific and sample specific. Different genes have different degradation extent, and different samples may have different average degradation extent.*

*For example, longer genes tend to degrade more than shorter genes. Even we could do a simulation by controlling a fixed proportion of sample to have degradation, each gene of which with fixed degree of degradation (without variation), the results will have no practical value for guiding the users, as the real data is totally different.*

*Secondly, degradation within the same condition may have more clustered pattern than between conditions (as shown in SEQC-AB and Simulation III), which may affects accuracy of the results dramatically.*

*In Simulation II and Simulation III, we already showed that each sample have the exactly same marginal distribution of degradation (SF5a,b) (average degradation and number of genes that have degradation are all same). But the accuracy assessed based on ROC curve from simulations is completely different. In this revision, we also tabulated the TPR rate at FPR threshold level 0.05 (Supplementary Table 3).*

*Likewise in Simulation IV, regardless of that marginal degradation of condition B was much worse than in Simulation II and III, results are much better than that simulation III. So accuracy of the results cannot be simply judged by the sample-wise degradation level. The condition specific degradation pattern could be much more influential on the results.*

*Thirdly, not only the absolute level degradation matters, but also the relative degradation extent and the homogeneity of degradation patter matter.*

*In the real data, for example, GBM R10 vs R4, and GBM R6 vs R4, we found the false positive rate in GBM R10 vs R4 is much higher than GBM R6 vs R4 regardless that the latter R6 samples have more degradation than R10. So the relative difference or homogeneity of degradation between samples make a larger difference than samplewise average degradation.*

*We also made a few naïve examples in our previous reply letter, which we think were quite illustrative of this point.*

*In summary we believe that our existing real data and simulation studies already sufficiently demonstrated how different factors may affect the accuracy of analysis. Even we could do simplified simulation by fixing proportion of genes that have degradation and fixing the level of degradation of gene, such results will not provide any meaningful help for users as the real data is way different from such naïve settings.*

4.The same holds true for the reply to “The authors should also benchmark their method using real clinical samples with known differential genes or pathways.” Only assessing false negatives is not good enough. As proposed originally, I invite the authors to also include data from (partially) degraded/fragmented RNA samples with prior knowledge on differential pathways or genes. For sure, these exist. One could look for studies in which a transcription factor was silenced or upregulated and combined with known info on transcriptional target genes or regulated pathways, the authors could point out that their method is as good or better to recapitulate what is known from a transcriptional perspective. Other examples are the use of targeted drugs with well understood transcriptional effects; or comparing different cell types, organs, cell cycle stages, gender, healthy vs. diseases samples, etc.

*First of all, we are confused by Reviewer’s comment “Only assessing false negatives is not good enough”. Throughout this paper, we never intended to solely address false negative rate. Our discussion always addresses both false positive and false negative if it applies (i.e. when there are both true nulls and true alternatives). If all nulls are true, we addressed false positives only as there are no false negatives. We would appreciate if the Reviewer could be more informative by pointing to specific contents in the text or letter. In the case we indeed had such careless error(s), we want to correct it.*

*We spent many hours searching for suitable set in clinical data in GEO data. Unfortunately most data are just similar. Unlike the SEQC-AB data, which was created in international consortium for benchmarking analysis, real clinical data is not created for this purpose. Instead RNA-seq is a tool used to identify possible differentially expressed genes, not to gauge different methods. PCR verification is done typically for a handful set of genes that are test-positives. Test-negative genes, which are typically not of interest, are rarely verified in such studies. Without verification of both sides, rigorously analysis of the performance of a method is impossible.*

*By “ground truth”, we require the status of expression of all genes or at least large set of genes. In this regard, we disagree with the Reviewer that the ground truth can be inferred or estimated based on knowledge. That’s why such studies still need to use qRT-PCR for verifications. We insist that the rigorous analysis of sensitivity (TPR) and specificity have to be grounded on large-scale verifications when the data contains both true positives and true negatives.*

*In the end we settled down on a data set published in Nature Cell Biology in 2018, which appears to satisfy Reviewer’s most criteria (but it still only has a small set of verified genes). This data set arose from a clinical study on how AMP kinase (AMPK) promotes glioblastoma bioenergetics and tumor growth [30]. It was shown that cancer cells can activate AMPK and highjack the stress-regulating pathway in cells. Thus inhibiting AMPK in cancer cells may lead to treatment of GBM. RNA-seq data was collected from two patient-derived GBM stem cell between control and AMPK knockout to identify differentially expressed genes. The results have been presented as a case study on page 8, 18, 19 and Supplementary Table 2 in this revision.*

**Reviewer 3:**

p. 10 "and a pairwise correlation matrix of DI scores between samples (Supplementary Figs. 1a-f)." Correlation would show that samples have a systematic gene-wise degradation regardless of overall quality of the degradation. I think given the argument that degradation can vary by gene, Euclidean distance makes more sense.

*We appreciate that the Reviewer provided this helpful and constructive suggestion. We generated the heatmap based on the Euclidean distance of DI scores. The pattern looks very similar for each figure (Please see Supplementary Fig. 7 in the uploaded file. But larger distance corresponds to small correlation in the color scheme). For this reason, we didn’t replace the current heatmap pictures.*

**Third round of review**

**Reviewer 2**

Although I still have questions, I believe the 2nd revised version of the manuscript is good enough so that the community can elaborate further on the concept and start using it. By using the method in additional real-life scenarios, the method will become ‘community validated’. 

In my 4th comment, I meant ‘false positives’, sorry. The additional data set is definitely useful. However, there are many more useful data sets. Such data-sets do not need to contain per se the truth or validation data (which I believe the authors have a hard time to find), but could simply be RNA seq data of experimental conditions that are well studied or validated (e.g. drug X upregulates pathway Y in disease Z according to e.g. MS protein analyses; well, RNA seq data on the use of drug X in disease Z could be used to confirm regulation of pathway Y). 

One final minor comment, at a few locations ‘qRT-PCR’ is used but it should be ‘RT-qPCR’ (according to MIQE guidelines).

**Reviewer 3**

The authors addressed my comment on correlation versus Euclidean distance adequately and I feel the paper is ready for publication. However, there are still some grammatical problems.
